# The stool microbiota of insulin resistant women with recent gestational diabetes, a high risk group for type 2 diabetes

**DOI:** 10.1038/srep13212

**Published:** 2015-08-17

**Authors:** Marina Fugmann, Michaela Breier, Marietta Rottenkolber, Friederike Banning, Uta Ferrari, Vanessa Sacco, Harald Grallert, Klaus G. Parhofer, Jochen Seissler, Thomas Clavel, Andreas Lechner

**Affiliations:** 1 Diabetes Research Group, Medizinische Klinik und Poliklinik IV, Klinikum der Universität München, Ludwig-Maximilians-Universität München, Munich, Germany; 2 Clinical Cooperation Group Type 2 Diabetes, Helmholtz Zentrum München, Munich, Germany; 3 German Center for Diabetes Research (DZD), Munich, Germany; 4 Research Unit of Molecular Epidemiology, Helmholtz Zentrum München, German Research Center for Environmental Health, Neuherberg, Germany; 5 Institute of Epidemiology II, Helmholtz Zentrum München, German Research Center for Environmental Health, Neuherberg, Germany; 6 Institute for Medical Information Sciences, Biometry and Epidemiology, Ludwig-Maximilians-Universität München, Munich, Germany; 7 Medizinische Klinik und Poliklinik II, Klinikum der Universität München, Ludwig-Maximilians-Universität München, Munich, Germany; 8 Junior Research Group Intestinal Microbiome, ZIEL-Research Center for Nutrition and Food Sciences, Technische Universität München, Freising-Weihenstephan, Germany

## Abstract

The gut microbiota has been linked to metabolic diseases. However, information on the microbiome of young adults at risk for type 2 diabetes (T2D) is lacking. The aim of this cross-sectional analysis was to investigate whether insulin resistant women with previous gestational diabetes (pGDM), a high risk group for T2D, differ in their stool microbiota from women after a normoglycemic pregnancy (controls). Bacterial communities were analyzed by high-throughput 16S rRNA gene sequencing using fecal samples from 42 pGDM and 35 control subjects 3–16 months after delivery. Clinical characterization included a 5-point OGTT, anthropometrics, clinical chemistry markers and a food frequency questionnaire. Women with a *Prevotellaceae*-dominated intestinal microbiome were overrepresented in the pGDM group (p < 0.0001). Additionally, the relative abundance of the phylum *Firmicutes* was significantly lower in women pGDM (median 48.5 vs. 56.8%; p = 0.013). Taxa richness (alpha diversity) was similar between the two groups and with correction for multiple testing we observed no significant differences on lower taxonomic levels. These results suggest that distinctive features of the intestinal microbiota are already present in young adults at risk for T2D and that further investigations of a potential pathophysiological role of gut bacteria in early T2D development are warranted.

Data from human and animal studies suggest that the gut microbiota influences metabolic health^1^,^2^. The microbiota itself can be markedly affected by dietary habits and medications as well as probably by other until now not fully defined factors^3^,^4^.

Several studies examined stool microbiota changes associated with metabolic diseases in human subjects. A lower bacterial diversity has been described in association with obesity and insulin resistance^5^,^6^. On the level of bacterial phyla, a decreased ratio of *Bacteroidetes* to *Firmicutes* (B/F ratio) has been shown to be associated with obesity^7^,^8^ but these findings are controversial^9^,^10^,^11^. Finucane *et al*. concluded in a recent review that there are no simple taxonomic compositions, consistent over different studies, which differentiate between obese and lean individuals^12^. Reduced relative abundance of *Firmicutes* and of the class *Clostridia* was found in individuals with type 2 diabetes (T2D)^13^,^14^,^15^. On a functional level, several groups reported a reduction of butyrate-producing bacteria in diabetic individuals^14^,^15^,^16^. A causal role of the gut microbiota in the development of T2D and also metabolic syndrome is supported by transplantation studies, both in animal models and in humans^17^,^18^.

Previous human studies concerning associations of the intestinal microbiota with T2D focused on individuals above 50 years of age^14^,^15^,^16^ and no information is available on the microbiome composition of younger subjects at risk for T2D. In this study, we therefore wanted to test if the composition of the stool microbiota already varied with T2D risk in young adults. Since no biomarkers exist to reliably identify at-risk subjects at this age we chose insulin resistant women with a recent history of gestational diabetes (GDM) as our high risk cohort and compared these to a suitable control group. Women post-GDM have a substantially increased risk for T2D, particularly if they remain insulin resistant after the pregnancy^19^,^20^,^21^.

## Results

### Baseline characteristics

We selected two diametrically opposed groups of women from a prospective post-gestation study: Insulin resistant women with a recent history of GDM (post-GDM/pGDM; n = 42) and women after a normoglycemic pregnancy as controls (n = 35). All data and samples were collected 3 to 16 months after delivery. The clinical baseline characteristics of the study cohort are shown in Table 1. A proportion of 50% of the women pGDM had impaired fasting glucose (IFG) and/or impaired glucose tolerance (IGT), whereas all controls were normoglycemic. Data from the EPIC food-frequency questionnaire^22^ (n = 64) showed no significant differences in dietary intake of macronutritients and fiber between the pGDM and the control group (Table S1).

### Bacterial community structure (beta diversity) in the two study groups

**Figure 1:**
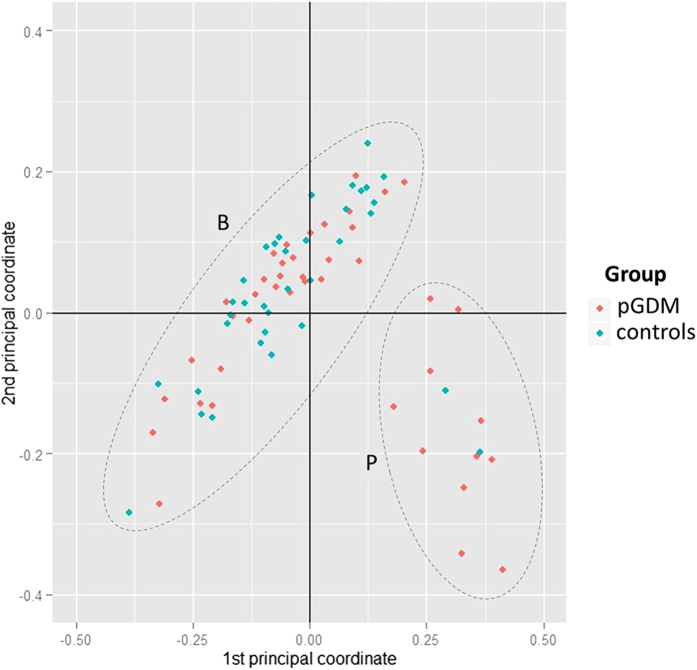
PCoA of the bacterial community composition based on Bray Curtis analysis. One dot represents one individual (post-GDM = red, controls = blue). pMANOVA revealed no significant differences between the prespecified groups (p = 0.1). However, two other clusters were identified in an analysis without prespecifications (designated clusters B and P; p = 0.001).

**Figure 2:**
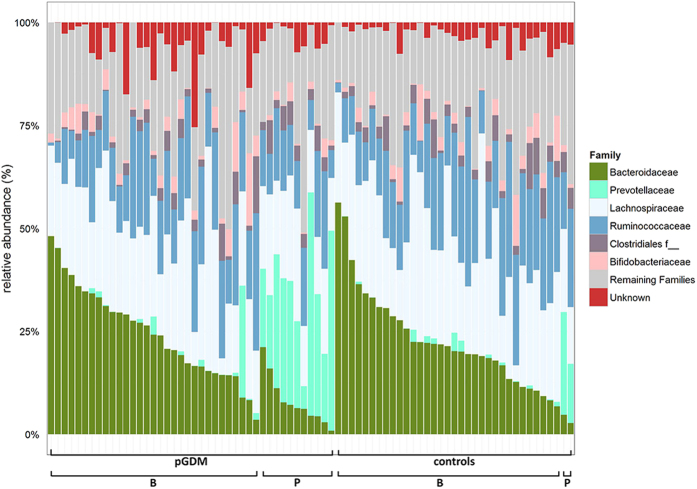
Top 6 bacterial families in the fecal microbiota. One bar represents one study participant. Subjects were first sorted by group (pGDM vs. control), then by cluster (Fig. 1) and then by the relative abundance of *Bacteroidaceae*. The category ‘remaining families’ includes all families except for the top 6. f__ = unknown family.

A total number of 307 Operational Taxonomic Units (OTUs) were quantified in this study. To examine the bacterial community structure in the stool samples, we analyzed the dataset by a principal coordinate analysis (PCoA) on the basis of Bray-Curtis distances and a permutational MANOVA (pMANOVA)^23^. We found no significant clustering of the predefined post-GDM and control group (p = 0.1). However, the samples from 13 women (cluster P) showed a bacterial composition distinct from the rest of the study cohort (cluster B) (Fig. 1, p = 0.001 in pMANOVA). The relative abundance of 35 OTUs was significantly different between these two groups after correction for multiple testing (Table S2). In particular, members of the family *Prevotellaceae* were more abundant in samples within cluster P (24.9 [17.8–30.2] vs. 0.08 [0.02–1.01] %, p < 0.0001), whereas the sequence proportion of the family *Bacteroidaceae* was decreased (6.2 [4.4–7.7] vs. 21.9 [15.9–30.8] %, p < 0.0001; Fig. 2). OTU2, which could be assigned down to the species level as *Prevotella copri*, was highly enriched in the subpopulation of cluster P (23.3 [13.1–28.5] vs. 0.01 [0.00–0.02] %, p < 0.001). Cluster P was significantly associated with belonging to the post-GDM group (11 out of 13 individuals in cluster P; p < 0.0001). EPIC-FFQ data revealed no differences in consumption of macronutrients and of different food groups (meat, fat, vegetables, fruits and cereals) between the women in cluster P and those in cluster B. A canonical correspondence analysis (CCA) and permutation tests showed no significant influence of age (p = 0.664), bmi (p = 0.806), time since delivery (p = 0.309) and plasma leptin levels (p = 0.294) on the beta diversity data.

### Phylum-level differences between the post-GDM and the control group

At the phylum level, *Actinobacteria, Bacteroidetes, Firmicutes, Proteobacteria* and *Verrucomicrobia* accounted on average for 95.4% of total sequences in all study participants, whereas 4.2% were unknown bacterial phyla. A minor fraction of sequences (0.4%) was assigned to the phyla *Euryarchaeota, Elusimicrobia, Fusobacteria* and *Lentisphaerae* (referred to as “others” in this manuscript).

**Figure 3:**
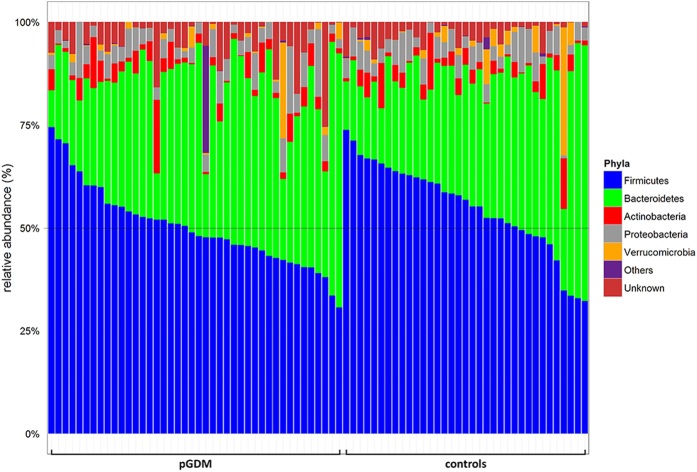
Relative abundances of bacterial phyla. Each bar represents one individual. Subjects were first sorted by group (pGDM vs. control), then by the relative abundance of *Firmicutes*, the most common phylum.

Group comparisons for phyla abundances are shown in Fig. 3 and Table S3. *Firmicutes* was less abundant in women post-GDM than in controls (48.5 [43.2–55.1] vs. 56.8 [48.5–63.2] %, p = 0.013). This finding remained unchanged by the variable ‘time since delivery’ in a bivariate regression analysis (data not shown). We also saw a reduced proportion of the phylum *Firmicutes* when we compared only the normoglycemic women post-GDM (n = 21) with the control group (n = 35; all normoglycemic) (50.5 [45.6–55.5] vs. 56.8 [48.–63.2] %, p = 0.045). The proportion of *Firmicutes* also remained lower in the pGDM group when we removed all women in cluster P from the analysis (p = 0.049).

### Bacterial richness (alpha diversity) and results on lower taxonomic levels

We found no differences in the alpha diversity between pGDM and control subjects (Fig. S1). Similarly, we observed no significant differences in the taxonomic ranks class, order, family and OTU between the two study groups when we applied corrections for multiple testing. These data and the exploratory (uncorrected) p-values are shown in (Table S4).

## Discussion

In this study, we compared the stool microbiota of a group of young adults at high risk for subsequent T2D, namely of insulin resistant women after recent GDM with those of a control group - women after a normoglycemic pregnancy. The main findings of this study were that the analysis of beta diversity separated the study participants into two clusters with distinct microbiome compositions and that the less common, *Prevotellaceae*-rich type of microbiome associated strongly with the post-GDM group. Additionally, at the phylum level, the proportion of *Firmicutes* was lower in the women post-GDM.

Identifying young adults at risk for T2D is difficult because of a lack of suitable biomarkers or highly predictive clinical parameters. Family history or a prediabetic glucose tolerance status have been used for this purpose but these approaches have significant limitations (discussed in^20^). We therefore used an alternative method to define a young adult at risk cohort and studied women with recent GDM, who remained insulin resistant after delivery. Women with GDM have an about 10-fold increased risk for T2D within 10 years compared to women normoglycemic during pregnancy^21^. Among those post GDM, this risk is further increased if insulin resistance persists after giving birth^19^,^20^.

Our analysis of beta diversity revealed that a subgroup of 13 women (17% of the study cohort) had a distinct microbiota composition characterized by a high proportion of the family *Prevotellaceae*, while *Bacteroidaceae* dominated in the other study participants. This finding was not explained by differences in dietary habits and is in line with a model of two enterotypes proposed recently^24^,^25^. This model remains controversial^26^ and our study lacks the sample size to settle the issue. Nevertheless, a *Prevotellaceae*-rich microbiome was associated with belonging to the high risk group for T2D in our study. Our finding adds to previous reports of an inverse association between the *Bacteroides*/*Prevotella ratio* and obesity^27^, non-alcoholic steatohepatitis^28^ and elevated total plasma cholesterol^25^. Potential causative links remain speculative. *Prevotella* are mucin degrading bacteria, which may be associated with increased gut permeability^29^ and *P. copri*, the most dominant molecular species within the taxa *Prevotella* in our study, has recently been associated with new-onset rheumatoid arthritis^30^. It could therefore be related to low-grade inflammation, which is also detrimental with respect to metabolism.

We further observed a lower relative abundance of *Firmicutes* in the post-GDM group, a finding previously reported for individuals with already established T2D^13^,^14^,^15^. This observation was also true for the normoglycemic women in our study and remained significant in an analysis restricted to women with a *Bacteroidaceae*-rich microbiome. The difference was small (median 49 vs. 57%) and a relevant overlap existed between the post-GDM and the control group (Fig. 3). Hence, the cause-effect relationships remain unknown. Higher resolution analyses on lower taxonomic levels, requiring large sample sizes, may nevertheless reveal specific underlying differences in microbial composition or function.

The main limitations of our present study are the limited sample size and the fact that only one stool sample per study participant was available. Additionally, the observational, cross-sectional study design precludes examining causality. Strengths of our work are the young adult, uniform cohorts of individuals with little concomitant medication and comorbidities and the detailed clinical phenotyping available.

In conclusion, this study suggests that distinctive features of the intestinal microbiota are already present in young adults at risk for T2D. In particular, it supports a link between a *Prevotellaceae*-dominated microbiome and T2D risk. Our results warrant further investigation in larger human cohorts and other clinical settings, as well as examination of the underlying molecular mechanisms.

## Subjects and Methods

### Study population

Subjects included in the present analysis were participants of the prospective, mono-center observational study PPS-Diab (prediction, prevention and subclassification of type 2 diabetes) enrolled between November 2011 and December 2013 as described previously^20^. Women with GDM during their last pregnancy and women following a normoglycemic pregnancy, treated at the Diabetes Center and the obstetrics department of the University Hospital (Klinikum der Universität München) in Munich, Germany, were consecutively recruited for this study in a 2:1 ratio. The diagnosis of GDM was based on a standardized oral glucose tolerance test (OGTT) after the 23rd week of the preceding pregnancy. Exclusion criteria for this study were alcohol or substance abuse, positivity for islet-autoantibodies and chronic diseases requiring medication except for hypothyroidism (n = 13). The study participants were not taking any antibiotic therapy for at least 14 days prior to clinical assessment and stool collection. The study was performed in accordance with relevant guidelines and regulations. Written informed consent was obtained from all study participants and the protocol was approved by the ethical review committee of the Ludwig-Maximilians-Universität München.

All data used in the present analysis were collected at the baseline visit of the PPS-Diab study, 3 to 16 months after the index pregnancy. For this analysis we selected two groups of women from the first 147 eligible study participants (thereof 96 post-GDM women with Matsuda Index = 4.2 [2.9–6.9]): the 45 most insulin resistant women with recent GDM (Matsuda Index = 3.3 [2.4–4.4]) and no diabetes at the time of the baseline visit (high risk subjects) and all 35 controls after a normoglycemic pregnancy (low risk subjects), from whom a stool sample was available. The samples of three individuals failed quality control after sequencing. The final study cohort therefore consisted of 42 high risk and 35 low risk women.

### Anthropometric and clinical assessments

All subjects underwent a 5-point OGTT and anthropometric measurements. To quantify insulin sensitivity the Matsuda Index was calculated from the OGTT plasma glucose and insulin measurements as described previously^31^ and the HOMA-IR was calculated as the product of fasting glucose and fasting insulin concentrations^32^. The disposition index was calculated from the OGTT to describe the relationship between insulin sensitivity and first-phase insulin secretion^33^. Thereby, the rise in serum insulin during the first 30 minutes of the OGTT was used as a measure of the first-phase insulin secretion. Prediabetes was defined as IFG, IGT or a combination of both following the definition of the American Diabetes Association^34^.

Systolic and diastolic blood pressure was measured twice in a sitting position. Weight and body fat mass was assessed by a bioelectrical impedance analysis (BIA) scale (Tanita BC-418, Tanita Corporation, Tokyo, Japan). The bmi was calculated as weight in kilograms divided by height squared in meters. Waist and hip circumference were measured with a tape measurement. A food frequency questionnaire (EPIC-FFQ) for the assessment of dietary nutritional intake was completed online (n = 64)^22^.

### Blood samples

Blood samples were processed immediately after collection and analyzed directly or stored in aliquots at −80 °C. Standard methods were used to measure glucose (glucose oxidase method, Glucose HK Gen.3, Roche Diagnostics, Mannheim, Germany), hba1c (VARIANT™ II TURBO HbA1c Kit - 2.0, Bio-Rad Laboratories, Hercules, USA), gamma-GT (enzymatic caloric test, Roche Diagnostics), triglycerides and HDL (enzymatic caloric test, Roche Diagnostics). LDL was calculated by the Friedewald equation (all triglyceride levels were below 400 mg/dl). Stored serum samples were used to measure insulin by chemiluminescence technology (CLIA, DiaSorin LIAISON systems, Saluggia, Italy). High-sensitive CRP (hsCRP) and leptin were measured from stored plasma samples by ELISAs (hsCRP by wide-range CRP, Siemens AG, Erlangen, Germany and leptin by ELISA “Dual Range”, Merck Millipore, Darmstadt, Germany).

### Stool sample collection

Each study participant collected one stool sample at home using a paper stool collector and tubes pre-filled with 8 ml of stool DNA stabilizer and including a measuring spoon for the sample (PSP Spin Stool DNA Plus Kit, STRATEC Molecular, Berlin, Germany). Samples were mailed to the study center within one day after collection and then immediately stored at −80 °C until DNA extraction.

### Microbiota sequencing

Bacterial DNA was obtained from fecal samples according to the manufacturer’s instructions (PSP Spin Stool DNA Plus Kit, STRATEC Molecular). The V4 region of 16S rRNA genes was amplified (25 cycles) as described previously^35^ following a 2-step procedure to limit artifacts^36^. Amplicons were purified using the AMPure XP system (Beckman Coulter GmbH, Krefeld, Germany) and sequenced in paired-end modus (PE200) using the MiSeq system (Illumina Inc., San Diego, USA). The raw read files were demultiplexed (allowing a maximum of 2 errors in barcodes) and each sample was processed using usearch^37^ following the UPARSE approach^38^. First, all reads were trimmed to the position of the first base with quality score <3 and then paired. The resulted sequences were size filtered, excluding those with assembled size <200 and >260. Paired reads with a number of expected error >3 were further filtered out and the remaining sequences were trimmed by 10 nucleotides on each side to avoid GC bias and non-random base composition. For each sample, sequences were de-replicated and checked for chimeras with UCHIME^39^. Sequences from all samples were merged, sorted by abundance and OTUs representatives were picked at a threshold of 97% similarity. Finally, all sequences were mapped back to the representative sequences resulting in the OTU table for all samples. The RDP classifier^40^ was used to assign taxonomic classification to the OTUs representative sequences (80% confidence). OTUs of particular interest were identified more precisely using the EzTaxon server: http://www.ezbiocloud.net/eztaxon^41^. A phylogenetic tree was constructed using fasttree^42^. Only OTUs with a relative abundance above 0.5% total sequences in at least one sample were kept. The OTU table was rarefied to the minimum count of sequences observed. After processing (quality- and chimera-check as well as OTU filtering) a total of 2,655,517 16S rRNA reads were used for analysis. We obtained a median of 33,807 sequences per sample with a range of 21,812–51,777 and used 20,000 sequences per sample for rarefaction.

### Statistical analysis

Statistical analyses were performed using R version 3.1.0^43^ and SPSS version 22.0 (SPSS Inc., Chicago, IL, USA). Clinical baseline characteristics are presented as median with interquartile ranges (Q1-Q3). Alpha diversity was measured by the Chao1 Index, which primary calculates the number of taxa^44^ and by the Shannon and Simpson Indices, which represent richness and distribution of taxa^45^. Differences in beta diversity were calculated by pMANOVA^46^ and illustrated by a PCoA of Bray Curtis distances^23^. The Chi-square test was used for comparing the two subtypes of microbiota composition identified by pMANOVA. A canonical correspondence analysis (CCA) and permutation tests were done using the R package phyloseq. The relative abundances of the taxonomic ranks phylum, class, order, family and of the molecular species are shown as median, interquartile ranges and minimum (min) and maximum (max). The Mann-Whitney U test, the Fisher Exact test or the χ-square test were used for comparisons between groups. The phylum *Firmicutes* was further analyzed together with the variable ‘time since delivery’ as independent variables by a binary logistic regression model for the dependent variable pGDM status. P-values <0.05 were considered statistically significant. Correction for multiple testing by false discovery rate control with the Benjamini–Hochberg procedure was done where indicated in the text.

## Additional Information

**How to cite this article**: Fugmann, M. *et al*. The stool microbiota of insulin resistant women with recent gestational diabetes, a high risk group for type 2 diabetes. *Sci. Rep*. **5**, 13212; doi: 10.1038/srep13212 (2015).

## Supplementary Material

Supplementary Information

## Figures and Tables

**Table 1:** Clinical and biochemical characteristics of pGDM and controls.

	pGDM n = 42	controls n = 35	p-value
clinical characteristics during previous pregnancy			
GDM with/without insulin	27/15 64.3%/35.7%	—	
clinical characteristics at postpartum study visit			
NGT/PGT(IFG/IGT/IFG + IGT)	21/21(7/10/4) 50%/50% (16.7%/23.8%/9.5%)	35 100%	
age (years)	37 [34–39]	36 [32–38]	0.242
months post delivery	8.8 [6.6–10.6]	8.8 [6.9–10.2]	0.914
breast feeding at time of visit (full/partial/no)	3/12/27 (7.1%/28.6%/64.3%)	0/15/20 (0%/42.9%/57.1%)	0.194^#^
primipara (yes/no)	19/23 (45.2%/54.8%)	19/16 (54.3%/45.7%)	0.429^##^
hormonal contraception (yes/no)	12/30 (28.6%/71.4%)	5/29 (14.3%/82.9%)	0.149^##^
regular menstrual cycle without hormonal contraception (yes/no)	19/10 (63.3%/33.3%)	12/17 (41.4%/58.6%)	0.065^##^
bmi (kg/m^2^)	27.0 [23.9–31.6]	22.6 [21.3–26.2]	<0.001
waist circumference (cm)	86.0 [77.0–99.0]	74.0 [70.0–86.0]	<0.001
whr	0.81 [0.77–0.87]	0.79 [0.74–0.83]	0.029
body fat_BIA_ (%) (n = 42/34)	36.4 [29.9–42.5]	30.3 [25.2–36.0]	0.002
systolic blood pressure (mmHg)	120 [111–130]	112 [105–123]	0.006
diastolic blood pressure (mmHg)	74 [67–81]	70 [64–79]	0.060
biochemical characteristics at postpartum study visit			
Matsuda Index	3.19 [2.27–4.35]	7.31 [4.84–9.21]	<0.001
HOMA-IR	2.65 [1.91–3.63]	1.22 [0.81–1.91]	<0.001
Disposition Index	176.04 [126.54–233.28]	309.52 [230.54–388.33]	<0.001
triglycerides (mg/dl)	89 [70–110]	63 [52–87]	0.001
hdl cholesterol (mg/dl)	55 [44–64]	64 [56–75]	<0.001
ldl cholesterol (mg/dl)	107 [91–124]	115 [97–133]	0.346
hsCRP (mg/dl)	0.13 [0.03–0.40]	0.06 [0.01–0.12]	0.003
gamma-glutamyl transferase (U/l)	18 [13–22]	13 [11–16]	0.003
hba1c (%) (n = 42/34)	5.4 [5.3–5.6]	5.3 [5.1–5.5]	0.060
leptin (ng/ml)	13.50 [9.36–19.73]	7.01 [2.48–12.28]	<0.001

Values are represented as median [Q1–Q3] or as n and %. Mann-Whitney U tests were used except for ^#^Fisher Exact test and ^##^χ-square test. NGT = normal glucose tolerance. PGT = pathologic glucose tolerance. IFG = impaired fasting glucose. IGT = impaired glucose tolerance.
