# Supplementary material for: The stool microbiota of insulin resistant women with recent gestational diabetes, a high risk group for type 2 diabetes
Source: Sci Rep. 2015 Aug 17;5:13212. doi: 10.1038/srep13212 (PMC4538691; doi:10.1038/srep13212)
Supplement: Supplementary Information [file srep13212-s1.doc]

**The stool microbiota of insulin resistant women with recent gestational diabetes, a high risk group for type 2 diabetes**

Marina Fugmann, Michaela Breier, Marietta Rottenkolber, Friederike Banning, Uta Ferrari, Vanessa Sacco, Harald Grallert, Klaus G. Parhofer, Jochen Seissler, Thomas Clavel, Andreas Lechner

**Supplementary Figure 1.** Alpha diversity in pGDM and controls (Chao1, Shannon and Simpson Index).

**
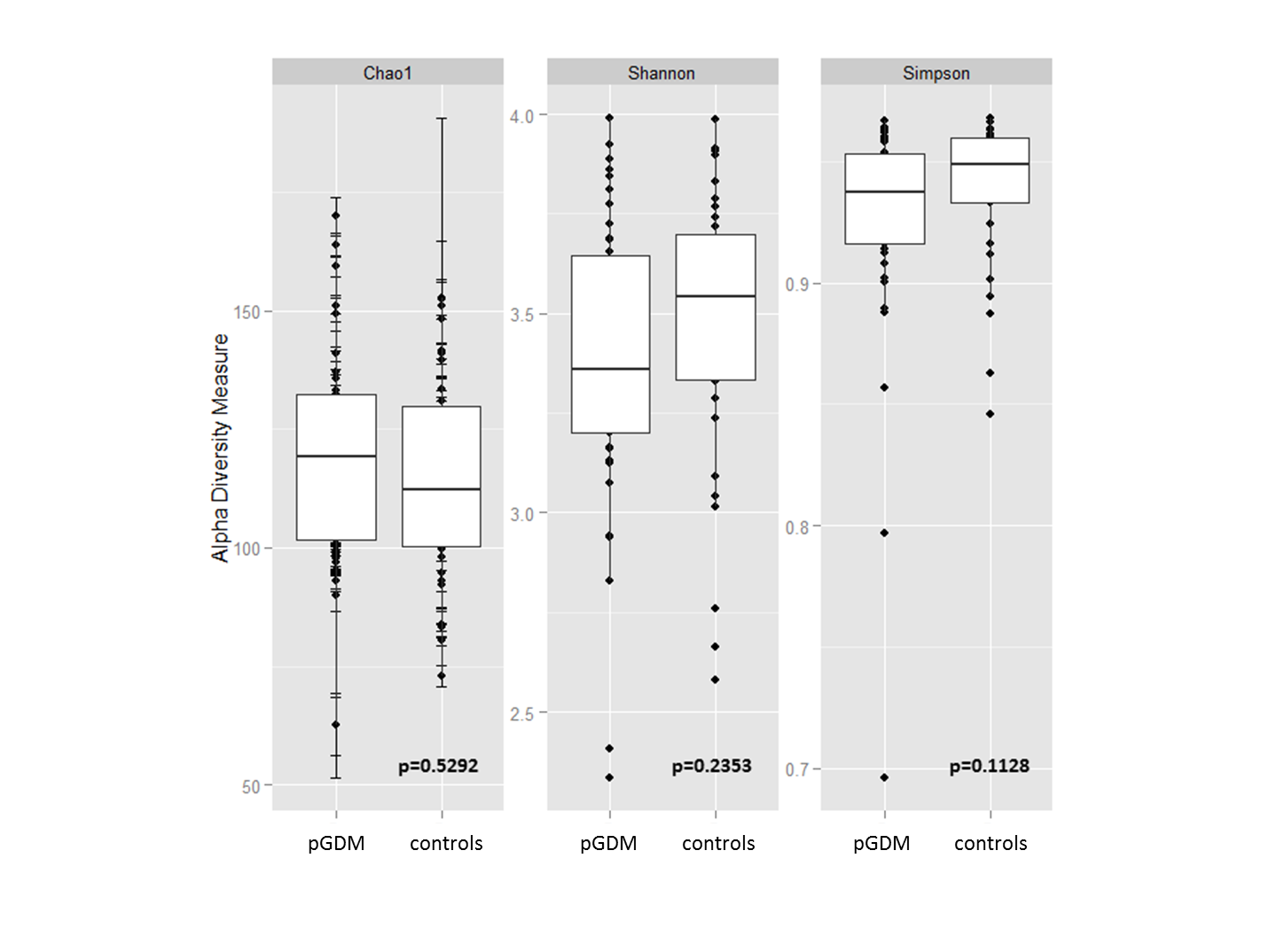
**

**Supplementary Table 1. Group comparisons of nutritional data determined by EPIC-FFQ.**

|  | **pGDM n=34** | **controls n=30** | **p-value** |
| --- | --- | --- | --- |
|  | **(Median [Q1-Q3])** | **(Median [Q1-Q3])** |  |
| **Total energy (kJ/day)** | 9052 (7386-10510) | 7812 (6610-10493) | 0.146 |
| **Fat (g/day)** | 97 (83-108) | 83 (68-117) | 0.085 |
| **Protein (g/day)** | 71 (64-83) | 65 (49-86) | 0.095 |
| **Carbohydrate (g/day)** | 240 (183-286) | 211 (184-277) | 0.563 |
| **Fibre (g/day)** | 24 (19-31) | 21 (17-27) | 0.276 |

**Supplementary Table 2.** Overview of 35 OTUs that were characteristic for 13 subjects with peculiar community structure after beta diversity analysis. OTU sequences were identified using EzTaxon[^37^](#_ENREF_37). The closest hit species is shown. Remaining OTUs that were insignificant are shown in **Table S4**.

|  |  | **EzTaxon** | **13 subjects** | | | | | |  | **64 subjects** | | | | | |
| --- | --- | --- | --- | --- | --- | --- | --- | --- | --- | --- | --- | --- | --- | --- | --- |
| **OTU** | ***Taxonomy*** | **Pairwise similarity (%)** | **n** | **Median** | **Q1** | **Q3** | **min** | **max** | **n** | **Median** | **Q1** | **Q3** | **min** | **max** | **p-value** |
| 1 | *Bacteroides dorei* and *vulgatus* | 99.57 | 13 | 2.25 | 1.24 | 2.98 | 0.34 | 9.12 | 64 | 8.91 | 4.71 | 14.88 | 0.04 | 41.55 | <0.001 |
| 15 | *Bacteroides ovatus* and *xylanisolvens* | 99.14 | 13 | 0.40 | 0.23 | 0.50 | 0.06 | 0.95 | 64 | 1.45 | 0.61 | 3.18 | 0.01 | 15.07 | <0.001 |
| 8 | *Bacteroides uniformis ^#^* | 99.57 | 13 | 0.51 | 0.32 | 0.71 | 0.06 | 3.02 | 64 | 2.47 | 1.44 | 6.35 | 0.02 | 14.73 | <0.001 |
| 42 | *Bacteroides xylanolyticus ^#^* | 98.28 | 13 | 2.54 | 1.32 | 2.95 | 0.55 | 5.80 | 62 | 0.41 | 0.14 | 2.25 | 0.00 | 6.88 | 0.003 |
| 14 | *Clostridium amygdalinum* | 97.00 | 3 | 0.00 | 0.00 | 0.00 | 0.00 | 0.04 | 47 | 0.04 | 0.00 | 0.17 | 0.00 | 12.25 | * |
| 184 | *Clostridium bolteae ^#^* | 100.00 | 6 | 0.00 | 0.00 | 0.02 | 0.00 | 0.03 | 56 | 0.04 | 0.01 | 0.12 | 0.00 | 0.86 | 0.001 |
| 382 | *Clostridium cellobioparum* | 90.13 | 12 | 0.14 | 0.07 | 0.34 | 0.00 | 2.45 | 23 | 0.00 | 0.00 | 0.05 | 0.00 | 2.49 | <0.001 |
| 239 | *Clostridium leptum* | 92.27 | 2 | 0.00 | 0.00 | 0.00 | 0.00 | 0.67 | 0 | 0.00 | 0.00 | 0.00 | 0.00 | 0.00 | * |
| 247 | *Clostridium symbiosum* | 83.26 | 8 | 0.01 | 0.00 | 0.19 | 0.00 | 0.56 | 6 | 0.00 | 0.00 | 0.00 | 0.00 | 0.16 | <0.001 |
| 72 | *Clostridium termitidis* | 92.27 | 13 | 0.47 | 0.27 | 1.10 | 0.02 | 3.12 | 41 | 0.05 | 0.00 | 0.46 | 0.00 | 3.72 | 0.004 |
| 27 | *Coprococcus eutactus ^#^* | 99.57 | 11 | 1.82 | 0.29 | 2.06 | 0.00 | 7.59 | 37 | 0.01 | 0.00 | 0.40 | 0.00 | 15.18 | 0.005 |
| 36 | *Dialister propionicifaciens ^#^* | 98.28 | 7 | 0.20 | 0.00 | 3.33 | 0.00 | 6.76 | 14 | 0.00 | 0.00 | 0.00 | 0.00 | 2.14 | 0.003 |
| 74 | *Eubacterium biforme ^#^* | 100.00 | 8 | 0.10 | 0.00 | 0.30 | 0.00 | 0.78 | 13 | 0.00 | 0.00 | 0.00 | 0.00 | 3.93 | 0.002 |
| 146 | *Eubacterium coprostanoligenes* | 95.28 | 8 | 0.10 | 0.00 | 0.24 | 0.00 | 0.79 | 15 | 0.00 | 0.00 | 0.00 | 0.00 | 2.01 | 0.005 |
| 31 | *Eubacterium coprostanoligenes* | 93.13 | 13 | 2.24 | 0.16 | 2.77 | 0.01 | 5.21 | 30 | 0.00 | 0.00 | 0.03 | 0.00 | 11.75 | <0.001 |
| 159 | *Eubacterium coprostanoligenes* | 90.95 | 11 | 0.12 | 0.02 | 0.45 | 0.00 | 1.07 | 15 | 0.00 | 0.00 | 0.00 | 0.00 | 1.20 | <0.001 |
| 221 | *Eubacterium desmolans ^#^* | 99.57 | 13 | 0.38 | 0.23 | 0.50 | 0.03 | 0.77 | 60 | 0.13 | 0.05 | 0.32 | 0.00 | 1.05 | 0.007 |
| 277 | *Eubacterium hallii ^#^* | 100.00 | 10 | 0.01 | 0.00 | 0.02 | 0.00 | 0.03 | 55 | 0.03 | 0.01 | 0.06 | 0.00 | 0.86 | 0.002 |
| 61 | *Eubacterium ruminantium* | 96.57 | 10 | 0.32 | 0.00 | 0.84 | 0.00 | 6.59 | 18 | 0.00 | 0.00 | 0.01 | 0.00 | 3.27 | 0.001 |
| 180 | *Faecalibacterium prausnitzii* | 94.42 | 8 | 0.03 | 0.00 | 0.07 | 0.00 | 1.68 | 7 | 0.00 | 0.00 | 0.00 | 0.00 | 0.51 | <0.001 |
| 158 | *Filifactor alocis* | 86.64 | 4 | 0.00 | 0.00 | 0.01 | 0.00 | 1.14 | 1 | 0.00 | 0.00 | 0.00 | 0.00 | 0.33 | * |
| 137 | *Flavonifractor plautii* | 94.42 | 5 | 0.00 | 0.00 | 0.01 | 0.00 | 0.04 | 48 | 0.08 | 0.00 | 0.34 | 0.00 | 1.39 | 0.002 |
| 151 | *Flavonifractor plautii ^#^* | 100.00 | 11 | 0.02 | 0.01 | 0.03 | 0.00 | 0.12 | 63 | 0.12 | 0.05 | 0.29 | 0.00 | 0.91 | <0.001 |
| 44 | *Fusicatenibacter saccharivorans ^#^* | 100.00 | 13 | 0.23 | 0.16 | 0.54 | 0.08 | 1.52 | 63 | 1.04 | 0.57 | 2.49 | 0.00 | 7.02 | 0.001 |
| 201 | *Hungatella hathewayi* | 97.00 | 5 | 0.00 | 0.00 | 0.21 | 0.00 | 0.78 | 3 | 0.00 | 0.00 | 0.00 | 0.00 | 0.02 | * |
| 258 | *Intestinibacter bartlettii ^#^* | 100.00 | 13 | 0.09 | 0.06 | 0.27 | 0.01 | 0.72 | 50 | 0.02 | 0.01 | 0.05 | 0.00 | 0.37 | <0.001 |
| 144 | *Oscillibacter ruminantium* | 94.85 | 13 | 0.03 | 0.01 | 0.05 | 0.01 | 0.39 | 62 | 0.29 | 0.10 | 0.52 | 0.00 | 1.64 | <0.001 |
| 190 | *Oscillibacter ruminantium* | 92.70 | 13 | 0.18 | 0.15 | 0.25 | 0.08 | 0.66 | 38 | 0.03 | 0.00 | 0.21 | 0.00 | 0.87 | 0.003 |
| 60 | *Oscillospira guilliermondii* | 93.13 | 13 | 1.21 | 0.58 | 2.03 | 0.05 | 4.58 | 47 | 0.16 | 0.00 | 1.16 | 0.00 | 3.31 | 0.005 |
| 250 | *Oscillospira guilliermondii* | 88.84 | 6 | 0.00 | 0.00 | 0.02 | 0.00 | 0.60 | 7 | 0.00 | 0.00 | 0.00 | 0.00 | 0.14 | 0.001 |
| 75 | *Prevotella copri* | 96.57 | 12 | 0.04 | 0.03 | 0.05 | 0.00 | 5.03 | 7 | 0.00 | 0.00 | 0.00 | 0.00 | 1.17 | <0.001 |
| 1215 | *Prevotella copri* | 95.28 | 8 | 0.02 | 0.00 | 0.08 | 0.00 | 3.20 | 8 | 0.00 | 0.00 | 0.00 | 0.00 | 10.44 | <0.001 |
| 2 | *Prevotella copri ^#^* | 97.85 | 13 | 23.27 | 13.14 | 28.47 | 3.46 | 54.32 | 38 | 0.01 | 0.00 | 0.02 | 0.00 | 2.12 | <0.001 |
| 1141 | *Prevotella copri ^#^* | 97.42 | 13 | 0.61 | 0.42 | 0.83 | 0.10 | 3.65 | 1 | 0.00 | 0.00 | 0.00 | 0.00 | 0.05 | * |
| 138 | *Prevotella shahii* | 90.13 | 3 | 0.00 | 0.00 | 0.00 | 0.00 | 1.52 | 0 | 0.00 | 0.00 | 0.00 | 0.00 | 0.00 | * |
| 1093 | *Prevotella stercorea ^#^* | 98.71 | 2 | 0.00 | 0.00 | 0.00 | 0.00 | 2.83 | 0 | 0.00 | 0.00 | 0.00 | 0.00 | 0.00 | * |
| 261 | [*Pseudoflavonifractor capillosus*](http://www.ezbiocloud.net/eztaxon/hierarchy?m=nomen_view&nid=Pseudoflavonifractor+capillosus) | 94.85 | 8 | 0.01 | 0.00 | 0.04 | 0.00 | 0.16 | 60 | 0.07 | 0.03 | 0.13 | 0.00 | 0.74 | 0.002 |
| 39 | *Roseburia faecis ^#^* | 98.28 | 13 | 0.07 | 0.04 | 0.56 | 0.01 | 3.22 | 43 | 0.02 | 0.00 | 0.08 | 0.00 | 7.15 | 0.004 |
| 142 | *Ruminococcus callidus ^#^* | 99.57 | 12 | 0.08 | 0.06 | 0.40 | 0.00 | 1.01 | 25 | 0.00 | 0.00 | 0.13 | 0.00 | 1.68 | 0.002 |
| 34 | *Ruminococcus gnavus ^#^* | 100.00 | 3 | 0.00 | 0.00 | 0.00 | 0.00 | 0.05 | 50 | 0.04 | 0.00 | 0.29 | 0.00 | 11.46 | * |
| 194 | *Spiroplasma culicicola* | 84.91 | 5 | 0.00 | 0.00 | 0.10 | 0.00 | 0.85 | 3 | 0.00 | 0.00 | 0.00 | 0.00 | 0.67 | * |
| 218 | *Sporobacter termitidis* | 94.42 | 12 | 0.17 | 0.08 | 0.25 | 0.00 | 0.91 | 39 | 0.01 | 0.00 | 0.07 | 0.00 | 0.55 | 0.001 |
| 172 | *Subdoligranulum variabile* | 94.85 | 10 | 0.02 | 0.00 | 0.03 | 0.00 | 0.05 | 57 | 0.05 | 0.01 | 0.12 | 0.00 | 1.22 | <0.001 |
| 29 | *Thermotalea metallivorans* and *Salimesophilobacter vulgaris* | 86.15 | 13 | 0.42 | 0.11 | 0.49 | 0.04 | 1.44 | 30 | 0.00 | 0.00 | 0.57 | 0.00 | 11.35 | 0.003 |

# OTUs that could be identified down to the species level, i.e., only one hit with >97.00% sequence similarity was identified, are marked with number sign. * p-values were not calculated when an OTU was present in less than 5 individuals in one of the two groups.

**Supplementary Table 3.** Comparison of relative sequence abundances at the different bacterial taxonomic ranks between pGDM and controls.

|  | **pGDM** | | | | | | **controls** | | | | | |  |
| --- | --- | --- | --- | --- | --- | --- | --- | --- | --- | --- | --- | --- | --- |
| **Phylum** | **n** | **Median** | **Q1** | **Q3** | **min** | **max** | **n** | **Median** | **Q1** | **Q3** | **min** | **max** | **p-value** |
| Firmicutes | 42 | 48.46 | 43.15 | 55.14 | 30.68 | 74.48 | 35 | 56.83 | 48.45 | 63.17 | 32.22 | 73.81 | 0.013 |
| Bacteroidetes | 42 | 36.57 | 25.66 | 40.81 | 8.93 | 61.71 | 35 | 29.83 | 20.82 | 36.17 | 11.78 | 62.09 | 0.091 |
| Proteobacteria | 42 | 3.66 | 3.04 | 5.90 | 1.62 | 16.44 | 35 | 4.73 | 3.12 | 6.84 | 0.65 | 10.95 | 0.431 |
| Actinobacteria | 42 | 2.31 | 1.10 | 5.09 | 0.21 | 17.90 | 35 | 2.61 | 0.93 | 4.01 | 0.20 | 13.80 | 0.667 |
| Verrucomicrobia | 33 | 0.43 | 0.02 | 1.45 | 0.00 | 23.27 | 28 | 1.05 | 0.06 | 3.24 | 0.01 | 31.13 | 0.144 |
| Others | 21 | 0.06 | 0.02 | 0.11 | 0.01 | 26.02 | 17 | 0.15 | 0.05 | 0.33 | 0.01 | 2.89 | 0.136 |
| Unknown Bacteria | 40 | 4.51 | 1.13 | 7.20 | 0.02 | 25.45 | 35 | 2.30 | 1.19 | 4.44 | 0.01 | 9.09 | 0.177 |
| Bacteroidetes/Firmicutes | 42 | 0.73 | 0.47 | 0.91 | 0.12 | 2.01 | 35 | 0.53 | 0.34 | 0.73 | 0.16 | 1.93 | 0.042 |
| **Class** |  |  |  |  |  |  |  |  |  |  |  |  |  |
| Euryarchaeota Methanobacteria | 9 | 0.02 | 0.02 | 0.12 | 0.01 | 0.30 | 6 | 0.27 | 0.17 | 0.34 | 0.01 | 0.63 | 0.066 |
| Bacteria (unknown) | 40 | 4.51 | 1.13 | 7.20 | 0.02 | 25.45 | 35 | 2.30 | 1.19 | 4.44 | 0.01 | 9.09 | 0.177 |
| Actinobacteria Actinobacteria | 42 | 2.31 | 1.10 | 5.09 | 0.21 | 17.90 | 35 | 2.61 | 0.93 | 4.01 | 0.20 | 13.80 | 0.667 |
| Bacteroidetes (unknown) | 31 | 0.23 | 0.04 | 1.43 | 0.01 | 8.51 | 25 | 0.50 | 0.11 | 1.40 | 0.01 | 6.77 | 0.627 |
| Bacteroidetes Bacteroidia | 42 | 35.54 | 24.61 | 40.65 | 7.48 | 61.60 | 35 | 29.34 | 19.52 | 36.17 | 10.57 | 62.09 | 0.091 |
| Elusimicrobia Elusimicrobia | 2 | 13.02 | 0.02 | 26.02 | 0.02 | 26.02 | 1 | 0.02 | 0.02 | 0.02 | 0.02 | 0.02 | * |
| Fusobacteria Fusobacteria | 1 | 0.08 | 0.08 | 0.08 | 0.08 | 0.08 | 1 | 2.79 | 2.79 | 2.79 | 2.79 | 2.79 | * |
| Lentisphaeria Lentisphaeria | 13 | 0.06 | 0.04 | 0.08 | 0.01 | 0.45 | 13 | 0.05 | 0.04 | 0.10 | 0.01 | 0.79 | 0.840 |
| Proteobacteria (unknown) | 17 | 0.13 | 0.03 | 0.57 | 0.00 | 1.07 | 13 | 0.06 | 0.02 | 0.55 | 0.01 | 3.76 | 0.902 |
| Proteobacteria Alphaproteobacteria | 41 | 1.58 | 0.71 | 2.39 | 0.02 | 12.58 | 34 | 1.59 | 0.77 | 3.97 | 0.01 | 8.47 | 0.798 |
| Proteobacteria Betaproteobacteria | 42 | 1.45 | 0.82 | 2.68 | 0.10 | 6.12 | 35 | 1.47 | 0.96 | 2.15 | 0.41 | 4.44 | 0.919 |
| Proteobacteria Deltaproteobacteria | 37 | 0.25 | 0.10 | 0.51 | 0.01 | 2.09 | 32 | 0.29 | 0.13 | 0.56 | 0.01 | 1.54 | 0.555 |
| Proteobacteria Gammaproteobacteria | 41 | 0.14 | 0.05 | 0.51 | 0.01 | 7.00 | 34 | 0.11 | 0.04 | 0.27 | 0.01 | 5.14 | 0.544 |
| Verrucomicrobia Opitutae | 8 | 0.27 | 0.20 | 0.38 | 0.07 | 0.47 | 8 | 0.17 | 0.01 | 0.41 | 0.00 | 1.93 | 0.505 |
| Verrucomicrobia Verrucomicrobiae | 32 | 0.26 | 0.02 | 1.37 | 0.00 | 23.27 | 27 | 1.01 | 0.04 | 3.03 | 0.01 | 31.13 | 0.117 |
| Firmicutes (unknown) | 37 | 1.20 | 0.50 | 3.66 | 0.00 | 13.18 | 32 | 1.18 | 0.15 | 4.42 | 0.01 | 16.04 | 0.791 |
| Firmicutes Bacilli | 40 | 0.13 | 0.05 | 0.25 | 0.01 | 1.32 | 35 | 0.08 | 0.05 | 0.17 | 0.01 | 2.25 | 0.279 |
| Firmicutes Clostridia | 42 | 41.83 | 36.76 | 48.78 | 20.87 | 69.31 | 35 | 47.30 | 41.63 | 58.89 | 28.84 | 65.24 | 0.014 |
| Firmicutes Erysipelotrichia | 28 | 0.03 | 0.01 | 0.32 | 0.00 | 3.91 | 14 | 0.03 | 0.01 | 0.57 | 0.00 | 0.72 | 0.823 |
| Firmicutes Negativicutes | 42 | 3.66 | 2.15 | 6.02 | 0.78 | 22.16 | 35 | 3.21 | 2.40 | 4.70 | 0.09 | 9.28 | 0.526 |
| **Order** |  |  |  |  |  |  |  |  |  |  |  |  |  |
| Euryarchaeota Methanobacteria Methanobacteriales | 9 | 0.02 | 0.02 | 0.12 | 0.01 | 0.30 | 6 | 0.27 | 0.17 | 0.34 | 0.01 | 0.63 | 0.066 |
| Bacteria | 40 | 4.51 | 1.13 | 7.20 | 0.02 | 25.45 | 35 | 2.30 | 1.19 | 4.44 | 0.01 | 9.09 | 0.177 |
| Actinobacteria Actinobacteria Bifidobacteriales | 41 | 2.17 | 0.72 | 4.59 | 0.14 | 17.56 | 35 | 2.61 | 0.72 | 3.69 | 0.08 | 13.66 | 0.743 |
| Actinobacteria Actinobacteria Coriobacteriales | 40 | 0.24 | 0.13 | 0.35 | 0.00 | 2.82 | 29 | 0.16 | 0.07 | 0.23 | 0.01 | 1.44 | 0.070 |
| Bacteroidetes (unknown) | 31 | 0.23 | 0.04 | 1.43 | 0.01 | 8.51 | 25 | 0.50 | 0.11 | 1.40 | 0.01 | 6.77 | 0.627 |
| Bacteroidetes Bacteroidia Bacteroidales | 42 | 35.54 | 24.61 | 40.65 | 7.48 | 61.60 | 35 | 29.34 | 19.52 | 36.17 | 10.57 | 62.09 | 0.091 |
| Elusimicrobia Elusimicrobia Elusimicrobiales | 2 | 13.02 | 0.02 | 26.02 | 0.02 | 26.02 | 1 | 0.02 | 0.02 | 0.02 | 0.02 | 0.02 | * |
| Fusobacteria Fusobacteria Fusobacteriales | 1 | 0.08 | 0.08 | 0.08 | 0.08 | 0.08 | 1 | 2.79 | 2.79 | 2.79 | 2.79 | 2.79 | * |
| Lentisphaeria Lentisphaeria Victivallales | 13 | 0.06 | 0.04 | 0.08 | 0.01 | 0.45 | 13 | 0.05 | 0.04 | 0.10 | 0.01 | 0.79 | 0.840 |
| Proteobacteria (unknown) | 17 | 0.13 | 0.03 | 0.57 | 0.00 | 1.07 | 13 | 0.06 | 0.02 | 0.55 | 0.01 | 3.76 | 0.902 |
| Proteobacteria Alphaproteobacteria (unknown) | 13 | 0.49 | 0.09 | 0.63 | 0.01 | 8.20 | 12 | 0.09 | 0.03 | 1.13 | 0.01 | 5.23 | 0.470 |
| Proteobacteria Alphaproteobacteria Rhizobiales | 41 | 1.44 | 0.54 | 2.25 | 0.01 | 12.58 | 34 | 1.50 | 0.69 | 2.97 | 0.01 | 8.01 | 0.750 |
| Proteobacteria Betaproteobacteria (unknown) | 6 | 0.57 | 0.26 | 0.95 | 0.16 | 1.34 | 5 | 1.45 | 0.01 | 2.14 | 0.01 | 2.18 | 0.662 |
| Proteobacteria Betaproteobacteria Burkholderiales | 41 | 1.45 | 0.74 | 2.68 | 0.10 | 6.12 | 35 | 1.11 | 0.87 | 1.99 | 0.07 | 4.44 | 0.549 |
| Proteobacteria Deltaproteobacteria Desulfovibrionales | 37 | 0.25 | 0.10 | 0.51 | 0.01 | 2.09 | 32 | 0.29 | 0.13 | 0.56 | 0.01 | 1.54 | 0.555 |
| Proteobacteria Gammaproteobacteria Enterobacteriales | 32 | 0.10 | 0.02 | 0.39 | 0.00 | 6.99 | 28 | 0.03 | 0.01 | 0.19 | 0.00 | 1.53 | 0.068 |
| Proteobacteria Gammaproteobacteria Pasteurellales | 31 | 0.03 | 0.01 | 0.13 | 0.00 | 1.11 | 21 | 0.05 | 0.02 | 0.13 | 0.01 | 5.11 | 0.648 |
| Proteobacteria Gammaproteobacteria Pseudomonadales | 24 | 0.01 | 0.01 | 0.02 | 0.00 | 0.23 | 20 | 0.02 | 0.01 | 0.09 | 0.00 | 1.24 | 0.138 |
| Verrucomicrobia Opitutae Puniceicoccales | 8 | 0.27 | 0.20 | 0.38 | 0.07 | 0.47 | 8 | 0.17 | 0.01 | 0.41 | 0.00 | 1.93 | 0.505 |
| Verrucomicrobia Verrucomicrobiae Verrucomicrobiales | 32 | 0.26 | 0.02 | 1.37 | 0.00 | 23.27 | 27 | 1.01 | 0.04 | 3.03 | 0.01 | 31.13 | 0.117 |
| Firmicutes (unknown) | 37 | 1.20 | 0.50 | 3.66 | 0.00 | 13.18 | 32 | 1.18 | 0.15 | 4.42 | 0.01 | 16.04 | 0.791 |
| Firmicutes Bacilli Lactobacillales | 40 | 0.13 | 0.05 | 0.25 | 0.01 | 1.32 | 35 | 0.08 | 0.05 | 0.17 | 0.01 | 2.25 | 0.279 |
| Firmicutes Clostridia (unknown) | 9 | 0.15 | 0.05 | 0.70 | 0.01 | 1.02 | 2 | 0.25 | 0.15 | 0.36 | 0.15 | 0.36 | * |
| Firmicutes Clostridia Clostridiales | 42 | 41.74 | 36.42 | 48.74 | 20.87 | 69.31 | 35 | 47.23 | 41.63 | 58.89 | 28.84 | 65.24 | 0.013 |
| Firmicutes Erysipelotrichia Erysipelotrichales | 28 | 0.03 | 0.01 | 0.32 | 0.00 | 3.91 | 14 | 0.03 | 0.01 | 0.57 | 0.00 | 0.72 | 0.823 |
| Firmicutes Negativicutes Selenomonadales | 42 | 3.66 | 2.15 | 6.02 | 0.78 | 22.16 | 35 | 3.21 | 2.40 | 4.70 | 0.09 | 9.28 | 0.526 |
| **Family** |  |  |  |  |  |  |  |  |  |  |  |  |  |
| Euryarchaeota Methanobacteria Methanobacteriales Methanobacteriaceae | 9 | 0.02 | 0.02 | 0.12 | 0.01 | 0.30 | 6 | 0.27 | 0.17 | 0.34 | 0.01 | 0.63 | 0.066 |
| Bacteria (unknown) | 40 | 4.51 | 1.13 | 7.20 | 0.02 | 25.45 | 35 | 2.30 | 1.19 | 4.44 | 0.01 | 9.09 | 0.177 |
| Actinobacteria Actinobacteria Bifidobacteriales Bifidobacteriaceae | 41 | 2.17 | 0.72 | 4.59 | 0.14 | 17.56 | 35 | 2.61 | 0.72 | 3.69 | 0.08 | 13.66 | 0.743 |
| Actinobacteria Actinobacteria Coriobacteriales Coriobacteriaceae | 40 | 0.24 | 0.13 | 0.35 | 0.00 | 2.82 | 29 | 0.16 | 0.07 | 0.23 | 0.01 | 1.44 | 0.070 |
| Bacteroidetes (unknown) | 31 | 0.23 | 0.04 | 1.43 | 0.01 | 8.51 | 25 | 0.50 | 0.11 | 1.40 | 0.01 | 6.77 | 0.627 |
| Bacteroidetes Bacteroidia Bacteroidales (unknown) | 5 | 0.72 | 0.70 | 1.09 | 0.01 | 9.95 | 3 | 0.02 | 0.01 | 0.04 | 0.01 | 0.04 | * |
| Bacteroidetes Bacteroidia Bacteroidales Porphyromonadaceae | 42 | 2.50 | 1.20 | 4.10 | 0.32 | 7.56 | 35 | 2.16 | 1.41 | 4.15 | 0.03 | 18.74 | 0.830 |
| Bacteroidetes Bacteroidia Bacteroidales Prevotellaceae | 35 | 0.94 | 0.03 | 19.00 | 0.01 | 54.28 | 26 | 0.19 | 0.02 | 1.35 | 0.00 | 24.93 | 0.075 |
| Bacteroidetes Bacteroidia Bacteroidales Rikenellaceae | 42 | 1.90 | 0.37 | 2.99 | 0.00 | 9.68 | 35 | 2.82 | 1.34 | 4.78 | 0.04 | 10.42 | 0.026 |
| Bacteroidetes Bacteroidia Bacteroidales Bacteroidaceae | 42 | 18.21 | 8.91 | 29.55 | 0.85 | 48.13 | 35 | 20.19 | 12.73 | 28.66 | 2.75 | 56.26 | 0.533 |
| Elusimicrobia Elusimicrobia Elusimicrobiales Elusimicrobiaceae | 2 | 13.02 | 0.02 | 26.02 | 0.02 | 26.02 | 1 | 0.02 | 0.02 | 0.02 | 0.02 | 0.02 | * |
| Fusobacteria Fusobacteria Fusobacteriales Fusobacteriaceae | 1 | 0.08 | 0.08 | 0.08 | 0.08 | 0.08 | 1 | 2.79 | 2.79 | 2.79 | 2.79 | 2.79 | * |
| Lentisphaerae Lentisphaeria Victivallales Victivallaceae | 13 | 0.06 | 0.04 | 0.08 | 0.01 | 0.45 | 13 | 0.05 | 0.04 | 0.10 | 0.01 | 0.79 | 0.840 |
| Proteobacteria (unknown) | 17 | 0.13 | 0.03 | 0.57 | 0.00 | 1.07 | 13 | 0.06 | 0.02 | 0.55 | 0.01 | 3.76 | 0.902 |
| Proteobacteria Alphaproteobacteria (unknown) | 13 | 0.49 | 0.09 | 0.63 | 0.01 | 8.20 | 12 | 0.09 | 0.03 | 1.13 | 0.01 | 5.23 | 0.470 |
| Proteobacteria Alphaproteobacteria Rhizobiales Hyphomicrobiaceae | 41 | 1.44 | 0.54 | 2.25 | 0.01 | 12.58 | 34 | 1.50 | 0.69 | 2.97 | 0.01 | 8.01 | 0.750 |
| Proteobacteria Betaproteobacteria (unknown) | 6 | 0.57 | 0.26 | 0.95 | 0.16 | 1.34 | 5 | 1.45 | 0.01 | 2.14 | 0.01 | 2.18 | 0.662 |
| Proteobacteria Betaproteobacteria Burkholderiales (unknown) | 11 | 0.76 | 0.16 | 2.13 | 0.01 | 3.34 | 9 | 0.37 | 0.03 | 0.42 | 0.01 | 0.95 | 0.067 |
| Proteobacteria Betaproteobacteria Burkholderiales Sutterellaceae | 38 | 1.26 | 0.46 | 2.18 | 0.04 | 6.12 | 35 | 1.06 | 0.80 | 1.88 | 0.02 | 4.44 | 0.842 |
| Proteobacteria Deltaproteobacteria Desulfovibrionales Desulfovibrionaceae | 37 | 0.25 | 0.10 | 0.51 | 0.01 | 2.09 | 32 | 0.29 | 0.13 | 0.56 | 0.01 | 1.54 | 0.555 |
| Proteobacteria Gammaproteobacteria Enterobacteriales Enterobacteriaceae | 32 | 0.10 | 0.02 | 0.39 | 0.00 | 6.99 | 28 | 0.03 | 0.01 | 0.19 | 0.00 | 1.53 | 0.068 |
| Proteobacteria Gammaproteobacteria Pasteurellales Pasteurellaceae | 31 | 0.03 | 0.01 | 0.13 | 0.00 | 1.11 | 21 | 0.05 | 0.02 | 0.13 | 0.01 | 5.11 | 0.648 |
| Proteobacteria Gammaproteobacteria Pseudomonadales Moraxellaceae | 24 | 0.01 | 0.01 | 0.02 | 0.00 | 0.23 | 20 | 0.02 | 0.01 | 0.09 | 0.00 | 1.24 | 0.138 |
| Verrucomicrobia Opitutae Puniceicoccales Puniceicoccaceae | 8 | 0.27 | 0.20 | 0.38 | 0.07 | 0.47 | 8 | 0.17 | 0.01 | 0.41 | 0.00 | 1.93 | 0.505 |
| Verrucomicrobia Verrucomicrobiae Verrucomicrobiales Verrucomicrobialeae | 32 | 0.26 | 0.02 | 1.37 | 0.00 | 23.27 | 27 | 1.01 | 0.04 | 3.03 | 0.01 | 31.13 | 0.117 |
| Firmicutes (unknown) | 37 | 1.20 | 0.50 | 3.66 | 0.00 | 13.18 | 32 | 1.18 | 0.15 | 4.42 | 0.01 | 16.04 | 0.791 |
| Firmicutes Bacilli Lactobacillales Lactobacillaceae | 2 | 0.04 | 0.01 | 0.08 | 0.01 | 0.08 | 2 | 1.05 | 0.01 | 2.10 | 0.01 | 2.10 | * |
| Firmicutes Bacilli Lactobacillales Streptococcaceae | 40 | 0.13 | 0.05 | 0.25 | 0.01 | 1.32 | 35 | 0.08 | 0.05 | 0.15 | 0.01 | 2.13 | 0.274 |
| Firmicutes Clostridia (unknown) | 9 | 0.15 | 0.05 | 0.70 | 0.01 | 1.02 | 2 | 0.25 | 0.15 | 0.36 | 0.15 | 0.36 | * |
| Firmicutes Clostridia Clostridiales (unknown) | 42 | 2.34 | 1.00 | 3.96 | 0.03 | 13.86 | 35 | 2.49 | 1.29 | 4.94 | 0.08 | 11.16 | 0.455 |
| Firmicutes Clostridia Clostridiales Clostridiaceae 1 | 16 | 0.03 | 0.02 | 0.09 | 0.01 | 0.66 | 18 | 0.02 | 0.01 | 0.04 | 0.00 | 0.31 | 0.211 |
| Firmicutes Clostridia Clostridiales Clostridiales Incertae Sedis XI | 3 | 0.01 | 0.01 | 0.74 | 0.01 | 0.74 | 1 | 0.01 | 0.01 | 0.01 | 0.01 | 0.01 | * |
| Firmicutes Clostridia Clostridiales Eubacteriaceae | 2 | 0.10 | 0.05 | 0.16 | 0.05 | 0.16 | 4 | 0.11 | 0.01 | 0.39 | 0.01 | 0.58 | * |
| Firmicutes Clostridia Clostridiales Lachnospiraceae | 42 | 21.97 | 17.37 | 26.93 | 4.01 | 54.45 | 35 | 23.41 | 18.96 | 30.43 | 3.90 | 54.08 | 0.205 |
| Firmicutes Clostridia Clostridiales Peptostreptococcaceae | 42 | 0.09 | 0.04 | 0.23 | 0.01 | 1.87 | 35 | 0.07 | 0.03 | 0.12 | 0.01 | 0.71 | 0.118 |
| Firmicutes Clostridia Clostridiales Ruminococcaceae | 42 | 14.59 | 11.23 | 23.22 | 0.71 | 33.34 | 35 | 20.47 | 12.55 | 23.61 | 2.28 | 41.16 | 0.141 |
| Firmicutes Erysipelotrichia Erysipelotrichales Erysipelotrichaceae | 28 | 0.03 | 0.01 | 0.32 | 0.00 | 3.91 | 14 | 0.03 | 0.01 | 0.57 | 0.00 | 0.72 | 0.823 |
| Firmicutes Negativicutes Selenomonadales Acidaminococcaceae | 33 | 0.88 | 0.10 | 2.08 | 0.01 | 12.24 | 26 | 2.41 | 0.07 | 4.35 | 0.01 | 7.36 | 0.240 |
| Firmicutes Negativicutes Selenomonadales Veillonellaceae | 42 | 2.23 | 0.19 | 5.50 | 0.01 | 19.65 | 35 | 1.01 | 0.03 | 3.09 | 0.01 | 9.26 | 0.008 |

*p-values were not calculated when an OTU was present in less than 5 individuals in one of the two groups.

**Supplementary Table 4. Comparison of the relative abundances of molecular species (OTUs) between pGDM and controls.**

|  |  | **pGDM** | | | | | | **controls** | | | | | |  |
| --- | --- | --- | --- | --- | --- | --- | --- | --- | --- | --- | --- | --- | --- | --- |
| **OTU** | **RDP Taxonomy** | **n** | **Median** | **Q1** | **Q3** | **min** | **max** | **n** | **Median** | **Q1** | **Q3** | **min** | **max** | **p-value** |
| 1 | Bacteroides | 42 | 8.15 | 2.97 | 13.25 | 0.04 | 41.55 | 35 | 8.22 | 4.16 | 13.63 | 0.77 | 33.74 | 0.519 |
| 2 | Prevotella | 31 | 0.02 | 0.01 | 3.46 | 0.00 | 54.32 | 20 | 0.01 | 0.00 | 0.03 | 0.00 | 23.86 | 0.029 |
| 3 | Akkermansia | 29 | 0.01 | 0.00 | 0.42 | 0.00 | 23.15 | 22 | 0.02 | 0.00 | 1.49 | 0.00 | 8.34 | 0.768 |
| 4 | Elusimicrobium | 2 | 0.00 | 0.00 | 0.00 | 0.00 | 25.72 | 1 | 0.00 | 0.00 | 0.00 | 0.00 | 0.01 | * |
| 5 | Roseburia | 38 | 0.23 | 0.10 | 1.20 | 0.00 | 5.43 | 34 | 0.38 | 0.09 | 1.67 | 0.00 | 26.43 | 0.382 |
| 6 | Lachnospiraceae; g__ | 41 | 0.50 | 0.12 | 1.13 | 0.00 | 15.13 | 34 | 0.48 | 0.31 | 0.86 | 0.00 | 3.93 | 0.775 |
| 7 | unknown | 3 | 0.00 | 0.00 | 0.00 | 0.00 | 25.15 | 1 | 0.00 | 0.00 | 0.00 | 0.00 | 0.03 | * |
| 8 | Bacteroides | 42 | 2.20 | 0.71 | 5.06 | 0.06 | 14.73 | 35 | 2.18 | 1.24 | 6.09 | 0.02 | 10.35 | 0.759 |
| 9 | unknown | 40 | 1.73 | 0.45 | 4.31 | 0.00 | 17.59 | 34 | 1.61 | 0.63 | 3.68 | 0.00 | 7.29 | 0.740 |
| 10 | Bacteroides | 15 | 0.00 | 0.00 | 0.03 | 0.00 | 13.68 | 8 | 0.00 | 0.00 | 0.00 | 0.00 | 8.72 | 0.134 |
| 11 | Dialister | 28 | 0.06 | 0.00 | 3.02 | 0.00 | 10.90 | 21 | 0.01 | 0.00 | 2.47 | 0.00 | 5.35 | 0.237 |
| 12 | Lachnospiraceae; g__ | 6 | 0.00 | 0.00 | 0.00 | 0.00 | 25.65 | 0 | 0.00 | 0.00 | 0.00 | 0.00 | 0.00 | * |
| 13 | Ruminococcaceae; g__ | 33 | 0.62 | 0.01 | 1.98 | 0.00 | 8.10 | 28 | 0.23 | 0.01 | 2.49 | 0.00 | 20.42 | 0.568 |
| 14 | Lachnospiraceae; g__ | 26 | 0.02 | 0.00 | 0.08 | 0.00 | 3.97 | 24 | 0.04 | 0.00 | 0.17 | 0.00 | 12.25 | 0.247 |
| 15 | Bacteroides | 42 | 0.94 | 0.41 | 2.30 | 0.06 | 15.07 | 35 | 1.27 | 0.47 | 2.81 | 0.01 | 6.93 | 0.747 |
| 16 | Lachnospiraceae; g__ | 33 | 0.55 | 0.01 | 1.80 | 0.00 | 12.22 | 30 | 0.41 | 0.01 | 1.33 | 0.00 | 14.69 | 0.862 |
| 17 | Bifidobacterium | 41 | 2.08 | 0.66 | 4.53 | 0.00 | 17.56 | 35 | 2.46 | 0.74 | 3.57 | 0.07 | 12.85 | 0.858 |
| 18 | Lachnospiraceae; g__ | 29 | 0.15 | 0.00 | 0.62 | 0.00 | 2.41 | 29 | 0.29 | 0.02 | 1.07 | 0.00 | 17.34 | 0.053 |
| 19 | Bacteroides | 16 | 0.00 | 0.00 | 0.02 | 0.00 | 9.01 | 10 | 0.00 | 0.00 | 0.01 | 0.00 | 9.40 | 0.310 |
| 20 | Bacteroides | 21 | 0.00 | 0.00 | 1.09 | 0.00 | 7.84 | 22 | 0.01 | 0.00 | 1.14 | 0.00 | 11.84 | 0.487 |
| 21 | Gemmiger | 41 | 1.45 | 0.45 | 2.30 | 0.00 | 12.86 | 34 | 1.48 | 0.52 | 2.98 | 0.00 | 8.09 | 0.806 |
| 22 | Faecalibacterium | 42 | 4.46 | 2.69 | 6.62 | 0.02 | 13.22 | 35 | 4.80 | 2.29 | 8.85 | 0.02 | 16.23 | 0.449 |
| 23 | Phascolarctobacterium | 24 | 0.01 | 0.00 | 0.32 | 0.00 | 3.51 | 22 | 0.02 | 0.00 | 2.86 | 0.00 | 7.55 | 0.188 |
| 24 | Bacteroides | 18 | 0.00 | 0.00 | 0.07 | 0.00 | 2.74 | 19 | 0.01 | 0.00 | 1.69 | 0.00 | 11.74 | 0.110 |
| 25 | Acidaminococcus | 11 | 0.00 | 0.00 | 0.00 | 0.00 | 12.28 | 3 | 0.00 | 0.00 | 0.00 | 0.00 | 1.06 | * |
| 26 | Bacteroidales; f__; g__ | 2 | 0.00 | 0.00 | 0.00 | 0.00 | 9.89 | 2 | 0.00 | 0.00 | 0.00 | 0.00 | 0.03 | * |
| 27 | Coprococcus | 28 | 0.08 | 0.00 | 1.41 | 0.00 | 7.59 | 20 | 0.01 | 0.00 | 0.17 | 0.00 | 15.18 | 0.159 |
| 28 | unknown | 4 | 0.00 | 0.00 | 0.00 | 0.00 | 7.34 | 3 | 0.00 | 0.00 | 0.00 | 0.00 | 1.26 | * |
| 29 | Firmicutes; c__; o__; f__; g__ | 21 | 0.00 | 0.00 | 0.42 | 0.00 | 11.35 | 22 | 0.01 | 0.00 | 1.44 | 0.00 | 8.57 | 0.266 |
| 30 | Ruminococcus | 35 | 0.34 | 0.01 | 1.23 | 0.00 | 10.75 | 31 | 0.27 | 0.07 | 1.31 | 0.00 | 6.77 | 0.980 |
| 31 | Firmicutes; c__; o__; f__; g__ | 27 | 0.03 | 0.00 | 1.21 | 0.00 | 6.49 | 16 | 0.00 | 0.00 | 0.02 | 0.00 | 11.75 | 0.031 |
| 32 | Ruminococcaceae; g__ | 40 | 0.47 | 0.21 | 1.23 | 0.00 | 6.59 | 31 | 0.34 | 0.03 | 1.10 | 0.00 | 8.27 | 0.373 |
| 33 | Megamonas | 1 | 0.00 | 0.00 | 0.00 | 0.00 | 8.20 | 1 | 0.00 | 0.00 | 0.00 | 0.00 | 0.02 | * |
| 34 | Lachnospiracea incertae sedis | 27 | 0.02 | 0.00 | 0.11 | 0.00 | 5.80 | 26 | 0.04 | 0.00 | 0.39 | 0.00 | 11.46 | 0.527 |
| 35 | Bacteroides | 9 | 0.00 | 0.00 | 0.00 | 0.00 | 9.24 | 8 | 0.00 | 0.00 | 0.00 | 0.00 | 3.17 | 0.729 |
| 36 | Dialister | 13 | 0.00 | 0.00 | 0.01 | 0.00 | 6.76 | 8 | 0.00 | 0.00 | 0.00 | 0.00 | 5.94 | 0.351 |
| 37 | Bacteroides | 21 | 0.01 | 0.00 | 0.09 | 0.00 | 1.36 | 21 | 0.06 | 0.00 | 0.53 | 0.00 | 8.69 | 0.146 |
| 38 | Alphaproteobacteria; o__; f__; g__ | 9 | 0.00 | 0.00 | 0.00 | 0.00 | 8.00 | 8 | 0.00 | 0.00 | 0.00 | 0.00 | 3.42 | 0.827 |
| 39 | Lachnospiraceae; g__ | 36 | 0.04 | 0.01 | 0.15 | 0.00 | 7.15 | 20 | 0.01 | 0.00 | 0.05 | 0.00 | 0.38 | 0.002 |
| 40 | Lachnospiracea incertae sedis | 36 | 0.76 | 0.05 | 2.13 | 0.00 | 10.82 | 30 | 0.72 | 0.02 | 2.32 | 0.00 | 10.44 | 0.943 |
| 41 | Bacteroides | 31 | 0.16 | 0.00 | 0.85 | 0.00 | 5.33 | 22 | 0.05 | 0.00 | 0.52 | 0.00 | 2.14 | 0.284 |
| 42 | Lachnospiraceae; g__ | 40 | 0.75 | 0.15 | 2.61 | 0.00 | 6.88 | 35 | 0.57 | 0.23 | 2.96 | 0.01 | 6.07 | 0.500 |
| 43 | Roseburia | 40 | 0.27 | 0.05 | 1.00 | 0.00 | 7.35 | 34 | 0.24 | 0.10 | 0.89 | 0.00 | 4.64 | 0.927 |
| 44 | Lachnospiraceae; g__ | 42 | 0.76 | 0.26 | 1.52 | 0.05 | 7.02 | 34 | 1.05 | 0.44 | 2.49 | 0.00 | 5.92 | 0.334 |
| 45 | Clostridium IV | 30 | 0.03 | 0.00 | 0.09 | 0.00 | 3.53 | 28 | 0.18 | 0.01 | 0.63 | 0.00 | 9.36 | 0.028 |
| 46 | Parasutterella | 33 | 0.06 | 0.01 | 0.35 | 0.00 | 6.23 | 30 | 0.09 | 0.02 | 0.97 | 0.00 | 4.56 | 0.453 |
| 47 | Lachnospiraceae; g__ | 38 | 0.15 | 0.08 | 0.21 | 0.00 | 6.21 | 30 | 0.18 | 0.02 | 0.32 | 0.00 | 3.87 | 0.705 |
| 48 | unknown | 8 | 0.00 | 0.00 | 0.00 | 0.00 | 5.94 | 12 | 0.00 | 0.00 | 0.07 | 0.00 | 2.21 | 0.179 |
| 49 | Alistipes | 33 | 0.07 | 0.01 | 0.47 | 0.00 | 5.76 | 29 | 0.24 | 0.04 | 0.67 | 0.00 | 2.33 | 0.177 |
| 50 | Bacteroides | 1 | 0.00 | 0.00 | 0.00 | 0.00 | 0.62 | 2 | 0.00 | 0.00 | 0.00 | 0.00 | 5.28 | * |
| 51 | Megasphaera | 5 | 0.00 | 0.00 | 0.00 | 0.00 | 5.40 | 2 | 0.00 | 0.00 | 0.00 | 0.00 | 0.62 | * |
| 52 | Parabacteroides | 36 | 0.64 | 0.13 | 1.79 | 0.00 | 3.83 | 29 | 0.71 | 0.01 | 1.22 | 0.00 | 3.44 | 0.648 |
| 53 | Escherichia/Shigella | 32 | 0.03 | 0.00 | 0.25 | 0.00 | 6.98 | 27 | 0.02 | 0.00 | 0.08 | 0.00 | 0.93 | 0.119 |
| 54 | Mitsuokella | 4 | 0.00 | 0.00 | 0.00 | 0.00 | 5.89 | 0 | 0.00 | 0.00 | 0.00 | 0.00 | 0.00 | * |
| 55 | Coprococcus | 12 | 0.00 | 0.00 | 0.01 | 0.00 | 4.51 | 10 | 0.00 | 0.00 | 0.01 | 0.00 | 4.91 | 0.979 |
| 56 | Ruminococcaceae; g__ | 18 | 0.00 | 0.00 | 0.03 | 0.00 | 4.08 | 15 | 0.00 | 0.00 | 0.06 | 0.00 | 8.43 | 0.941 |
| 57 | Alistipes | 34 | 0.28 | 0.01 | 1.70 | 0.00 | 3.73 | 29 | 1.04 | 0.01 | 2.53 | 0.00 | 5.72 | 0.304 |
| 58 | Clostridiales; f__; g__ | 3 | 0.00 | 0.00 | 0.00 | 0.00 | 0.41 | 5 | 0.00 | 0.00 | 0.00 | 0.00 | 4.66 | * |
| 59 | Prevotella | 8 | 0.00 | 0.00 | 0.00 | 0.00 | 15.99 | 0 | 0.00 | 0.00 | 0.00 | 0.00 | 0.00 | * |
| 60 | Clostridiales; f__; g__ | 34 | 0.53 | 0.01 | 1.55 | 0.00 | 4.58 | 26 | 0.20 | 0.00 | 1.03 | 0.00 | 3.31 | 0.360 |
| 61 | Lachnospiraceae; g__ | 18 | 0.00 | 0.00 | 0.08 | 0.00 | 6.59 | 10 | 0.00 | 0.00 | 0.01 | 0.00 | 3.24 | 0.222 |
| 62 | Bacteroidetes; c__; o__; f__; g__ | 0 | 0.00 | 0.00 | 0.00 | 0.00 | 0.00 | 1 | 0.00 | 0.00 | 0.00 | 0.00 | 4.32 | * |
| 63 | Pasteurellaceae; g__ | 31 | 0.02 | 0.00 | 0.07 | 0.00 | 1.11 | 21 | 0.01 | 0.00 | 0.07 | 0.00 | 5.36 | 0.357 |
| 64 | Bacteroides | 39 | 0.23 | 0.07 | 0.47 | 0.00 | 2.12 | 33 | 0.29 | 0.11 | 0.67 | 0.00 | 4.67 | 0.321 |
| 65 | Collinsella | 39 | 0.22 | 0.10 | 0.35 | 0.00 | 2.86 | 28 | 0.10 | 0.02 | 0.21 | 0.00 | 1.43 | 0.044 |
| 66 | Dialister | 2 | 0.00 | 0.00 | 0.00 | 0.00 | 4.23 | 1 | 0.00 | 0.00 | 0.00 | 0.00 | 0.01 | * |
| 67 | Clostridiales; f__; g__ | 3 | 0.00 | 0.00 | 0.00 | 0.00 | 0.01 | 1 | 0.00 | 0.00 | 0.00 | 0.00 | 4.20 | * |
| 68 | Faecalibacterium | 42 | 1.66 | 0.90 | 3.70 | 0.02 | 10.56 | 35 | 2.39 | 0.77 | 3.50 | 0.06 | 7.01 | 0.988 |
| 69 | Sutterella | 22 | 0.01 | 0.00 | 1.07 | 0.00 | 4.21 | 22 | 0.14 | 0.00 | 0.95 | 0.00 | 2.69 | 0.561 |
| 70 | Prevotella | 3 | 0.00 | 0.00 | 0.00 | 0.00 | 4.18 | 1 | 0.00 | 0.00 | 0.00 | 0.00 | 4.53 | * |
| 71 | Bacteroidetes; c__; o__; f__; g__ | 2 | 0.00 | 0.00 | 0.00 | 0.00 | 0.04 | 1 | 0.00 | 0.00 | 0.00 | 0.00 | 6.71 | * |
| 72 | Clostridiales; f__; g__ | 32 | 0.19 | 0.00 | 0.64 | 0.00 | 3.72 | 22 | 0.15 | 0.00 | 0.43 | 0.00 | 2.09 | 0.189 |
| 73 | Lachnospiracea incertae sedis | 34 | 0.09 | 0.02 | 0.25 | 0.00 | 2.87 | 31 | 0.08 | 0.02 | 0.55 | 0.00 | 4.37 | 0.348 |
| 74 | Erysipelotrichaceae incertae sedis | 14 | 0.00 | 0.00 | 0.10 | 0.00 | 3.93 | 7 | 0.00 | 0.00 | 0.00 | 0.00 | 0.72 | 0.175 |
| 75 | Prevotella | 17 | 0.00 | 0.00 | 0.03 | 0.00 | 5.03 | 2 | 0.00 | 0.00 | 0.00 | 0.00 | 0.04 | * |
| 76 | Acidaminococcaceae; g__ | 9 | 0.00 | 0.00 | 0.00 | 0.00 | 4.10 | 4 | 0.00 | 0.00 | 0.00 | 0.00 | 4.44 | * |
| 77 | Burkholderiales; f__; g__ | 6 | 0.00 | 0.00 | 0.00 | 0.00 | 3.32 | 2 | 0.00 | 0.00 | 0.00 | 0.00 | 0.14 | * |
| 78 | Clostridiales; f__; g__ | 16 | 0.00 | 0.00 | 0.07 | 0.00 | 1.17 | 13 | 0.00 | 0.00 | 0.11 | 0.00 | 4.26 | 0.860 |
| 79 | Acidaminococcaceae; g__ | 3 | 0.00 | 0.00 | 0.00 | 0.00 | 3.34 | 3 | 0.00 | 0.00 | 0.00 | 0.00 | 5.07 | * |
| 80 | Clostridiales; f__; g__ | 9 | 0.00 | 0.00 | 0.00 | 0.00 | 3.50 | 1 | 0.00 | 0.00 | 0.00 | 0.00 | 0.01 | * |
| 81 | unknown | 1 | 0.00 | 0.00 | 0.00 | 0.00 | 2.93 | 0 | 0.00 | 0.00 | 0.00 | 0.00 | 0.00 | * |
| 82 | Ruminococcaceae; g__ | 35 | 0.08 | 0.03 | 0.33 | 0.00 | 3.22 | 27 | 0.13 | 0.00 | 0.43 | 0.00 | 1.68 | 0.651 |
| 83 | Ruminococcaceae; g__ | 5 | 0.00 | 0.00 | 0.00 | 0.00 | 3.31 | 2 | 0.00 | 0.00 | 0.00 | 0.00 | 0.71 | * |
| 84 | Proteobacteria; c__; o__; f__; g__ | 13 | 0.00 | 0.00 | 0.01 | 0.00 | 0.73 | 11 | 0.00 | 0.00 | 0.01 | 0.00 | 3.77 | 0.771 |
| 85 | Fusobacterium | 1 | 0.00 | 0.00 | 0.00 | 0.00 | 0.10 | 1 | 0.00 | 0.00 | 0.00 | 0.00 | 2.86 | * |
| 86 | Lachnospiraceae; g__ | 21 | 0.00 | 0.00 | 0.02 | 0.00 | 5.00 | 23 | 0.01 | 0.00 | 0.02 | 0.00 | 0.80 | 0.532 |
| 87 | Barnesiella | 28 | 0.09 | 0.00 | 0.81 | 0.00 | 3.22 | 23 | 0.02 | 0.00 | 0.79 | 0.00 | 2.49 | 0.794 |
| 88 | Firmicutes; c__; o__; f__; g__ | 28 | 0.24 | 0.00 | 0.43 | 0.00 | 1.33 | 22 | 0.18 | 0.00 | 0.42 | 0.00 | 3.47 | 0.587 |
| 89 | Prevotella | 2 | 0.00 | 0.00 | 0.00 | 0.00 | 2.59 | 0 | 0.00 | 0.00 | 0.00 | 0.00 | 0.00 | * |
| 90 | Bacteroides | 1 | 0.00 | 0.00 | 0.00 | 0.00 | 2.36 | 2 | 0.00 | 0.00 | 0.00 | 0.00 | 4.72 | * |
| 91 | Bacteroidetes; c__; o__; f__; g__ | 2 | 0.00 | 0.00 | 0.00 | 0.00 | 4.00 | 0 | 0.00 | 0.00 | 0.00 | 0.00 | 0.00 | * |
| 92 | Lachnospiraceae; g__ | 38 | 0.34 | 0.14 | 1.02 | 0.00 | 3.21 | 30 | 0.36 | 0.02 | 0.65 | 0.00 | 1.72 | 0.359 |
| 93 | Coprococcus | 36 | 0.37 | 0.12 | 0.71 | 0.00 | 3.76 | 29 | 0.27 | 0.07 | 0.43 | 0.00 | 1.40 | 0.184 |
| 94 | Ruminococcaceae; g__ | 25 | 0.03 | 0.00 | 0.43 | 0.00 | 2.67 | 18 | 0.02 | 0.00 | 0.38 | 0.00 | 3.73 | 0.613 |
| 95 | Lachnospiraceae; g__ | 39 | 0.48 | 0.12 | 0.74 | 0.00 | 3.35 | 29 | 0.29 | 0.11 | 0.49 | 0.00 | 2.03 | 0.241 |
| 96 | Megasphaera | 1 | 0.00 | 0.00 | 0.00 | 0.00 | 0.01 | 4 | 0.00 | 0.00 | 0.00 | 0.00 | 3.74 | * |
| 97 | Clostridiales; f__; g__ | 13 | 0.00 | 0.00 | 0.01 | 0.00 | 3.37 | 8 | 0.00 | 0.00 | 0.00 | 0.00 | 2.83 | 0.597 |
| 98 | Porphyromonadaceae; g__ | 0 | 0.00 | 0.00 | 0.00 | 0.00 | 0.00 | 1 | 0.00 | 0.00 | 0.00 | 0.00 | 6.07 | * |
| 99 | Bacteroidetes; c__; o__; f__; g__ | 1 | 0.00 | 0.00 | 0.00 | 0.00 | 3.67 | 0 | 0.00 | 0.00 | 0.00 | 0.00 | 0.00 | * |
| 100 | Clostridiales; f__; g__ | 5 | 0.00 | 0.00 | 0.00 | 0.00 | 1.93 | 5 | 0.00 | 0.00 | 0.00 | 0.00 | 2.72 | 0.503 |
| 101 | Bacteroidetes; c__; o__; f__; g__ | 18 | 0.00 | 0.00 | 0.09 | 0.00 | 2.32 | 19 | 0.00 | 0.00 | 0.35 | 0.00 | 3.16 | 0.619 |
| 102 | Sutterella | 7 | 0.00 | 0.00 | 0.00 | 0.00 | 1.76 | 7 | 0.00 | 0.00 | 0.00 | 0.00 | 3.05 | 0.882 |
| 103 | Bacteroidetes; c__; o__; f__; g__ | 2 | 0.00 | 0.00 | 0.00 | 0.00 | 2.09 | 0 | 0.00 | 0.00 | 0.00 | 0.00 | 0.00 | * |
| 104 | Clostridiales; f__; g__ | 3 | 0.00 | 0.00 | 0.00 | 0.00 | 0.91 | 10 | 0.00 | 0.00 | 0.00 | 0.00 | 3.42 | * |
| 105 | Desulfovibrio | 14 | 0.00 | 0.00 | 0.02 | 0.00 | 2.13 | 5 | 0.00 | 0.00 | 0.00 | 0.00 | 1.47 | 0.082 |
| 106 | Clostridiales; f__; g__ | 40 | 0.26 | 0.12 | 0.44 | 0.00 | 2.91 | 34 | 0.29 | 0.09 | 0.47 | 0.00 | 2.02 | 0.701 |
| 107 | Clostridiales; f__; g__ | 13 | 0.00 | 0.00 | 0.11 | 0.00 | 1.70 | 6 | 0.00 | 0.00 | 0.00 | 0.00 | 1.74 | 0.172 |
| 108 | unknown | 1 | 0.00 | 0.00 | 0.00 | 0.00 | 2.35 | 0 | 0.00 | 0.00 | 0.00 | 0.00 | 0.00 | * |
| 109 | Dorea | 38 | 0.27 | 0.13 | 0.44 | 0.00 | 1.75 | 33 | 0.28 | 0.04 | 0.64 | 0.00 | 1.97 | 0.782 |
| 110 | Ruminococcaceae; g__ | 30 | 0.09 | 0.00 | 0.53 | 0.00 | 2.97 | 19 | 0.01 | 0.00 | 0.30 | 0.00 | 1.43 | 0.178 |
| 111 | unknown | 11 | 0.00 | 0.00 | 0.01 | 0.00 | 2.09 | 11 | 0.00 | 0.00 | 0.04 | 0.00 | 0.65 | 0.937 |
| 112 | Firmicutes; c__; o__; f__; g__ | 0 | 0.00 | 0.00 | 0.00 | 0.00 | 0.00 | 1 | 0.00 | 0.00 | 0.00 | 0.00 | 2.74 | * |
| 113 | Firmicutes; c__; o__; f__; g__ | 7 | 0.00 | 0.00 | 0.00 | 0.00 | 0.27 | 10 | 0.00 | 0.00 | 0.01 | 0.00 | 2.14 | 0.157 |
| 114 | Lachnospiraceae; g__ | 17 | 0.00 | 0.00 | 0.05 | 0.00 | 1.92 | 12 | 0.00 | 0.00 | 0.03 | 0.00 | 0.33 | 0.384 |
| 115 | Burkholderiales; f__; g__ | 4 | 0.00 | 0.00 | 0.00 | 0.00 | 2.12 | 2 | 0.00 | 0.00 | 0.00 | 0.00 | 0.40 | * |
| 116 | Prevotella | 5 | 0.00 | 0.00 | 0.00 | 0.00 | 0.79 | 4 | 0.00 | 0.00 | 0.00 | 0.00 | 2.90 | * |
| 117 | Mitsuokella | 3 | 0.00 | 0.00 | 0.00 | 0.00 | 1.92 | 1 | 0.00 | 0.00 | 0.00 | 0.00 | 0.22 | * |
| 118 | Veillonella | 30 | 0.01 | 0.00 | 0.04 | 0.00 | 0.45 | 24 | 0.02 | 0.00 | 0.06 | 0.00 | 3.62 | 0.728 |
| 119 | Ruminococcus | 6 | 0.00 | 0.00 | 0.00 | 0.00 | 1.06 | 3 | 0.00 | 0.00 | 0.00 | 0.00 | 2.16 | * |
| 120 | Clostridiales; f__; g__ | 22 | 0.01 | 0.00 | 0.02 | 0.00 | 0.10 | 27 | 0.03 | 0.00 | 0.14 | 0.00 | 2.27 | 0.006 |
| 121 | Peptostreptococcaceae; g__ | 40 | 0.04 | 0.02 | 0.10 | 0.00 | 1.85 | 30 | 0.03 | 0.01 | 0.08 | 0.00 | 0.30 | 0.198 |
| 122 | Lachnospiraceae; g__ | 4 | 0.00 | 0.00 | 0.00 | 0.00 | 1.16 | 2 | 0.00 | 0.00 | 0.00 | 0.00 | 3.08 | * |
| 123 | Cerasicoccus | 8 | 0.00 | 0.00 | 0.00 | 0.00 | 0.49 | 8 | 0.00 | 0.00 | 0.00 | 0.00 | 1.94 | 0.790 |
| 124 | Burkholderiales; f__; g__ | 3 | 0.00 | 0.00 | 0.00 | 0.00 | 2.05 | 5 | 0.00 | 0.00 | 0.00 | 0.00 | 0.93 | * |
| 125 | Clostridiales; f__; g__ | 2 | 0.00 | 0.00 | 0.00 | 0.00 | 1.87 | 0 | 0.00 | 0.00 | 0.00 | 0.00 | 0.00 | * |
| 126 | Firmicutes; c__; o__; f__; g__ | 15 | 0.00 | 0.00 | 0.16 | 0.00 | 1.54 | 13 | 0.00 | 0.00 | 0.05 | 0.00 | 0.86 | 0.928 |
| 127 | Bacteroidetes; c__; o__; f__; g__ | 0 | 0.00 | 0.00 | 0.00 | 0.00 | 0.00 | 1 | 0.00 | 0.00 | 0.00 | 0.00 | 2.82 | * |
| 128 | Bacteroidetes; c__; o__; f__; g__ | 0 | 0.00 | 0.00 | 0.00 | 0.00 | 0.00 | 1 | 0.00 | 0.00 | 0.00 | 0.00 | 2.93 | * |
| 129 | Lachnospiraceae; g__ | 0 | 0.00 | 0.00 | 0.00 | 0.00 | 0.00 | 1 | 0.00 | 0.00 | 0.00 | 0.00 | 1.82 | * |
| 130 | Lachnospiraceae; g__ | 29 | 0.07 | 0.00 | 0.20 | 0.00 | 2.66 | 21 | 0.07 | 0.00 | 0.22 | 0.00 | 1.57 | 0.630 |
| 131 | Lachnospiraceae; g__ | 19 | 0.00 | 0.00 | 0.11 | 0.00 | 0.81 | 17 | 0.00 | 0.00 | 0.22 | 0.00 | 1.14 | 0.571 |
| 132 | unknown | 9 | 0.00 | 0.00 | 0.00 | 0.00 | 0.37 | 11 | 0.00 | 0.00 | 0.08 | 0.00 | 1.06 | 0.191 |
| 133 | unknown | 3 | 0.00 | 0.00 | 0.00 | 0.00 | 2.19 | 2 | 0.00 | 0.00 | 0.00 | 0.00 | 0.78 | * |
| 134 | Firmicutes; c__; o__; f__; g__ | 8 | 0.00 | 0.00 | 0.00 | 0.00 | 2.25 | 5 | 0.00 | 0.00 | 0.00 | 0.00 | 0.15 | 0.515 |
| 135 | unknown | 1 | 0.00 | 0.00 | 0.00 | 0.00 | 0.03 | 4 | 0.00 | 0.00 | 0.00 | 0.00 | 1.62 | * |
| 136 | Firmicutes; c__; o__; f__; g__ | 4 | 0.00 | 0.00 | 0.00 | 0.00 | 0.26 | 6 | 0.00 | 0.00 | 0.00 | 0.00 | 1.86 | * |
| 137 | Ruminococcaceae; g__ | 30 | 0.02 | 0.00 | 0.22 | 0.00 | 1.39 | 23 | 0.07 | 0.00 | 0.43 | 0.00 | 1.02 | 0.513 |
| 138 | Prevotella | 3 | 0.00 | 0.00 | 0.00 | 0.00 | 1.52 | 0 | 0.00 | 0.00 | 0.00 | 0.00 | 0.00 | * |
| 139 | Alphaproteobacteria; o__; f__; g__ | 1 | 0.00 | 0.00 | 0.00 | 0.00 | 0.05 | 3 | 0.00 | 0.00 | 0.00 | 0.00 | 1.88 | * |
| 140 | Lachnospiraceae; g__ | 26 | 0.02 | 0.00 | 0.13 | 0.00 | 0.80 | 25 | 0.09 | 0.00 | 0.35 | 0.00 | 1.84 | 0.082 |
| 141 | Odoribacter | 40 | 0.23 | 0.12 | 0.37 | 0.00 | 1.22 | 32 | 0.30 | 0.14 | 0.41 | 0.00 | 1.37 | 0.468 |
| 142 | Ruminococcus | 25 | 0.04 | 0.00 | 0.25 | 0.00 | 1.68 | 12 | 0.00 | 0.00 | 0.15 | 0.00 | 0.99 | 0.049 |
| 143 | Blautia | 42 | 0.18 | 0.09 | 0.46 | 0.03 | 1.56 | 35 | 0.30 | 0.18 | 0.59 | 0.08 | 1.90 | 0.016 |
| 144 | Oscillibacter | 41 | 0.20 | 0.03 | 0.43 | 0.00 | 1.16 | 34 | 0.30 | 0.10 | 0.52 | 0.00 | 1.64 | 0.183 |
| 145 | Clostridium IV | 17 | 0.00 | 0.00 | 0.04 | 0.00 | 1.82 | 15 | 0.00 | 0.00 | 0.07 | 0.00 | 1.58 | 0.628 |
| 146 | Clostridiales; f__; g__ | 13 | 0.00 | 0.00 | 0.06 | 0.00 | 1.08 | 10 | 0.00 | 0.00 | 0.05 | 0.00 | 2.01 | 0.742 |
| 147 | Proteobacteria; c__; o__; f__; g__ | 1 | 0.00 | 0.00 | 0.00 | 0.00 | 1.09 | 1 | 0.00 | 0.00 | 0.00 | 0.00 | 1.54 | * |
| 148 | Ruminococcus | 16 | 0.00 | 0.00 | 0.12 | 0.00 | 1.33 | 6 | 0.00 | 0.00 | 0.00 | 0.00 | 0.67 | 0.088 |
| 149 | Anaerostipes | 41 | 0.08 | 0.03 | 0.17 | 0.00 | 1.18 | 34 | 0.11 | 0.06 | 0.26 | 0.00 | 1.97 | 0.089 |
| 150 | Blautia | 23 | 0.01 | 0.00 | 0.03 | 0.00 | 0.49 | 18 | 0.01 | 0.00 | 0.05 | 0.00 | 2.54 | 0.746 |
| 151 | Flavonifractor | 39 | 0.09 | 0.02 | 0.22 | 0.00 | 0.91 | 35 | 0.11 | 0.04 | 0.28 | 0.02 | 0.87 | 0.216 |
| 152 | Bacteroidetes; c__; o__; f__; g__ | 8 | 0.00 | 0.00 | 0.00 | 0.00 | 1.08 | 3 | 0.00 | 0.00 | 0.00 | 0.00 | 1.35 | * |
| 153 | Enterobacteriaceae; g__ | 1 | 0.00 | 0.00 | 0.00 | 0.00 | 0.36 | 3 | 0.00 | 0.00 | 0.00 | 0.00 | 1.51 | * |
| 154 | Clostridiales; f__; g__ | 1 | 0.00 | 0.00 | 0.00 | 0.00 | 1.19 | 1 | 0.00 | 0.00 | 0.00 | 0.00 | 0.01 | * |
| 155 | Paraprevotella | 12 | 0.00 | 0.00 | 0.06 | 0.00 | 1.06 | 3 | 0.00 | 0.00 | 0.00 | 0.00 | 0.66 | * |
| 156 | unknown | 8 | 0.00 | 0.00 | 0.00 | 0.00 | 0.37 | 8 | 0.00 | 0.00 | 0.00 | 0.00 | 1.17 | 0.650 |
| 157 | Ruminococcaceae; g__ | 6 | 0.00 | 0.00 | 0.00 | 0.00 | 1.70 | 3 | 0.00 | 0.00 | 0.00 | 0.00 | 0.04 | * |
| 158 | Clostridiales; f__; g__ | 5 | 0.00 | 0.00 | 0.00 | 0.00 | 1.14 | 0 | 0.00 | 0.00 | 0.00 | 0.00 | 0.00 | * |
| 159 | Ruminococcaceae; g__ | 16 | 0.00 | 0.00 | 0.12 | 0.00 | 1.20 | 10 | 0.00 | 0.00 | 0.01 | 0.00 | 1.17 | 0.517 |
| 160 | Acinetobacter | 24 | 0.01 | 0.00 | 0.01 | 0.00 | 0.25 | 20 | 0.00 | 0.00 | 0.03 | 0.00 | 1.33 | 0.763 |
| 161 | Bacteroidetes; c__Bacteroidia; o__Bacteroidales; f__; g__ | 2 | 0.00 | 0.00 | 0.00 | 0.00 | 1.05 | 0 | 0.00 | 0.00 | 0.00 | 0.00 | 0.00 | * |
| 162 | Ruminococcus | 2 | 0.00 | 0.00 | 0.00 | 0.00 | 0.25 | 5 | 0.00 | 0.00 | 0.00 | 0.00 | 1.37 | * |
| 163 | Lactobacillus | 2 | 0.00 | 0.00 | 0.00 | 0.00 | 0.08 | 2 | 0.00 | 0.00 | 0.00 | 0.00 | 2.07 | * |
| 164 | Bilophila | 36 | 0.14 | 0.03 | 0.28 | 0.00 | 0.70 | 31 | 0.18 | 0.08 | 0.42 | 0.00 | 0.97 | 0.195 |
| 165 | Bacteroidetes; c__; o__; f__; g__ | 2 | 0.00 | 0.00 | 0.00 | 0.00 | 0.94 | 0 | 0.00 | 0.00 | 0.00 | 0.00 | 0.00 | * |
| 166 | Lachnospiraceae; g__ | 1 | 0.00 | 0.00 | 0.00 | 0.00 | 0.06 | 4 | 0.00 | 0.00 | 0.00 | 0.00 | 2.24 | * |
| 167 | Lachnospiraceae; g__ | 2 | 0.00 | 0.00 | 0.00 | 0.00 | 3.00 | 2 | 0.00 | 0.00 | 0.00 | 0.00 | 0.40 | * |
| 168 | Parabacteroides | 37 | 0.21 | 0.07 | 0.47 | 0.00 | 1.53 | 31 | 0.22 | 0.11 | 0.52 | 0.00 | 2.03 | 0.854 |
| 169 | Ruminococcaceae; g__ | 3 | 0.00 | 0.00 | 0.00 | 0.00 | 1.13 | 3 | 0.00 | 0.00 | 0.00 | 0.00 | 1.40 | * |
| 170 | Lachnospiraceae; g__ | 1 | 0.00 | 0.00 | 0.00 | 0.00 | 1.01 | 0 | 0.00 | 0.00 | 0.00 | 0.00 | 0.00 | * |
| 171 | Betaproteobacteria; o__; f__; g__ | 6 | 0.00 | 0.00 | 0.00 | 0.00 | 1.33 | 5 | 0.00 | 0.00 | 0.00 | 0.00 | 2.15 | 0.960 |
| 172 | Ruminococcaceae; g__ | 38 | 0.02 | 0.01 | 0.06 | 0.00 | 0.52 | 29 | 0.06 | 0.02 | 0.13 | 0.00 | 1.22 | 0.047 |
| 173 | Ruminococcaceae; g__ | 1 | 0.00 | 0.00 | 0.00 | 0.00 | 1.34 | 0 | 0.00 | 0.00 | 0.00 | 0.00 | 0.00 | * |
| 174 | Lachnospiraceae; g__ | 9 | 0.00 | 0.00 | 0.00 | 0.00 | 0.84 | 12 | 0.00 | 0.00 | 0.03 | 0.00 | 0.39 | 0.167 |
| 175 | Alistipes | 29 | 0.08 | 0.00 | 0.28 | 0.00 | 0.73 | 28 | 0.24 | 0.02 | 0.49 | 0.00 | 1.15 | 0.025 |
| 176 | Porphyromonadaceae; g__ | 0 | 0.00 | 0.00 | 0.00 | 0.00 | 0.00 | 2 | 0.00 | 0.00 | 0.00 | 0.00 | 1.49 | * |
| 177 | Clostridiales; f__; g__ | 1 | 0.00 | 0.00 | 0.00 | 0.00 | 1.54 | 2 | 0.00 | 0.00 | 0.00 | 0.00 | 0.89 | * |
| 178 | Bacteroidetes; c__; o__; f__; g__ | 1 | 0.00 | 0.00 | 0.00 | 0.00 | 0.09 | 1 | 0.00 | 0.00 | 0.00 | 0.00 | 0.98 | * |
| 179 | Ruminococcaceae; g__ | 1 | 0.00 | 0.00 | 0.00 | 0.00 | 0.82 | 0 | 0.00 | 0.00 | 0.00 | 0.00 | 0.00 | * |
| 180 | Faecalibacterium | 10 | 0.00 | 0.00 | 0.00 | 0.00 | 0.51 | 5 | 0.00 | 0.00 | 0.00 | 0.00 | 1.68 | 0.300 |
| 181 | Paraprevotella | 14 | 0.00 | 0.00 | 0.07 | 0.00 | 1.58 | 10 | 0.00 | 0.00 | 0.01 | 0.00 | 1.24 | 0.575 |
| 182 | Streptococcus | 38 | 0.07 | 0.03 | 0.16 | 0.00 | 1.30 | 34 | 0.05 | 0.03 | 0.13 | 0.00 | 1.40 | 0.506 |
| 183 | Clostridium IV | 7 | 0.00 | 0.00 | 0.00 | 0.00 | 0.96 | 3 | 0.00 | 0.00 | 0.00 | 0.00 | 1.08 | * |
| 184 | Clostridium XlVa | 32 | 0.03 | 0.00 | 0.09 | 0.00 | 0.42 | 30 | 0.04 | 0.01 | 0.12 | 0.00 | 0.86 | 0.243 |
| 185 | Lachnospiraceae; g__ | 39 | 0.28 | 0.12 | 0.44 | 0.00 | 1.32 | 30 | 0.24 | 0.06 | 0.73 | 0.00 | 1.25 | 0.914 |
| 186 | Ruminococcus | 3 | 0.00 | 0.00 | 0.00 | 0.00 | 1.25 | 0 | 0.00 | 0.00 | 0.00 | 0.00 | 0.00 | * |
| 187 | Blautia | 41 | 0.08 | 0.04 | 0.21 | 0.00 | 0.87 | 34 | 0.14 | 0.05 | 0.37 | 0.00 | 0.98 | 0.333 |
| 188 | Bacteroides | 3 | 0.00 | 0.00 | 0.00 | 0.00 | 1.08 | 1 | 0.00 | 0.00 | 0.00 | 0.00 | 0.03 | * |
| 189 | Bacteroidetes; c__; o__; f__; g__ | 4 | 0.00 | 0.00 | 0.00 | 0.00 | 1.43 | 1 | 0.00 | 0.00 | 0.00 | 0.00 | 0.43 | * |
| 190 | Oscillibacter | 32 | 0.10 | 0.01 | 0.21 | 0.00 | 0.79 | 19 | 0.03 | 0.00 | 0.25 | 0.00 | 0.87 | 0.119 |
| 191 | Sporobacter | 13 | 0.00 | 0.00 | 0.01 | 0.00 | 0.04 | 15 | 0.00 | 0.00 | 0.02 | 0.00 | 0.88 | 0.261 |
| 192 | Alphaproteobacteria; o__; f__; g__ | 6 | 0.00 | 0.00 | 0.00 | 0.00 | 0.66 | 5 | 0.00 | 0.00 | 0.00 | 0.00 | 1.20 | 0.933 |
| 193 | Flavonifractor | 38 | 0.09 | 0.03 | 0.21 | 0.00 | 0.76 | 34 | 0.15 | 0.06 | 0.20 | 0.00 | 0.38 | 0.449 |
| 194 | unknown | 6 | 0.00 | 0.00 | 0.00 | 0.00 | 0.67 | 2 | 0.00 | 0.00 | 0.00 | 0.00 | 0.85 | * |
| 195 | Paraprevotella | 1 | 0.00 | 0.00 | 0.00 | 0.00 | 0.56 | 2 | 0.00 | 0.00 | 0.00 | 0.00 | 0.83 | * |
| 196 | Clostridiales; f__; g__ | 6 | 0.00 | 0.00 | 0.00 | 0.00 | 1.20 | 3 | 0.00 | 0.00 | 0.00 | 0.00 | 1.06 | * |
| 197 | Ruminococcaceae; g__ | 42 | 0.12 | 0.06 | 0.25 | 0.01 | 0.60 | 34 | 0.08 | 0.06 | 0.23 | 0.00 | 0.75 | 0.591 |
| 198 | Ruminococcaceae; g__ | 36 | 0.04 | 0.02 | 0.09 | 0.00 | 1.47 | 27 | 0.04 | 0.01 | 0.10 | 0.00 | 0.40 | 0.708 |
| 199 | Firmicutes; c__; o__; f__; g__ | 12 | 0.00 | 0.00 | 0.01 | 0.00 | 1.08 | 8 | 0.00 | 0.00 | 0.00 | 0.00 | 0.15 | 0.720 |
| 200 | Lachnospiraceae; g__ | 24 | 0.01 | 0.00 | 0.07 | 0.00 | 0.70 | 22 | 0.05 | 0.00 | 0.15 | 0.00 | 0.83 | 0.222 |
| 201 | Lachnospiraceae; g__ | 7 | 0.00 | 0.00 | 0.00 | 0.00 | 0.78 | 1 | 0.00 | 0.00 | 0.00 | 0.00 | 0.57 | * |
| 202 | Lachnospiraceae; g__ | 22 | 0.00 | 0.00 | 0.12 | 0.00 | 0.64 | 20 | 0.02 | 0.00 | 0.12 | 0.00 | 0.43 | 0.754 |
| 203 | Clostridiales; f__; g__ | 0 | 0.00 | 0.00 | 0.00 | 0.00 | 0.00 | 1 | 0.00 | 0.00 | 0.00 | 0.00 | 1.06 | * |
| 204 | Clostridiales; f__; g__ | 2 | 0.00 | 0.00 | 0.00 | 0.00 | 0.05 | 3 | 0.00 | 0.00 | 0.00 | 0.00 | 1.06 | * |
| 205 | Bacteroides | 0 | 0.00 | 0.00 | 0.00 | 0.00 | 0.00 | 1 | 0.00 | 0.00 | 0.00 | 0.00 | 1.13 | * |
| 206 | Parvimonas | 3 | 0.00 | 0.00 | 0.00 | 0.00 | 0.68 | 1 | 0.00 | 0.00 | 0.00 | 0.00 | 0.03 | * |
| 207 | Alistipes | 11 | 0.00 | 0.00 | 0.01 | 0.00 | 0.86 | 10 | 0.00 | 0.00 | 0.01 | 0.00 | 0.97 | 0.734 |
| 208 | Ruminococcaceae; g__ | 24 | 0.01 | 0.00 | 0.05 | 0.00 | 0.74 | 18 | 0.01 | 0.00 | 0.11 | 0.00 | 0.46 | 0.847 |
| 209 | Bacteroides | 10 | 0.00 | 0.00 | 0.00 | 0.00 | 0.62 | 9 | 0.00 | 0.00 | 0.01 | 0.00 | 1.19 | 0.892 |
| 210 | Ruminococcus | 2 | 0.00 | 0.00 | 0.00 | 0.00 | 0.12 | 5 | 0.00 | 0.00 | 0.00 | 0.00 | 0.86 | * |
| 211 | Porphyromonadaceae; g__ | 0 | 0.00 | 0.00 | 0.00 | 0.00 | 0.00 | 1 | 0.00 | 0.00 | 0.00 | 0.00 | 3.07 | * |
| 212 | Clostridia; o__; f__; g__ | 3 | 0.00 | 0.00 | 0.00 | 0.00 | 1.04 | 0 | 0.00 | 0.00 | 0.00 | 0.00 | 0.00 | * |
| 213 | Firmicutes; c__; o__; f__; g__ | 3 | 0.00 | 0.00 | 0.00 | 0.00 | 0.47 | 2 | 0.00 | 0.00 | 0.00 | 0.00 | 0.69 | * |
| 214 | Ruminococcaceae; g__ | 1 | 0.00 | 0.00 | 0.00 | 0.00 | 0.76 | 0 | 0.00 | 0.00 | 0.00 | 0.00 | 0.00 | * |
| 215 | unknown | 0 | 0.00 | 0.00 | 0.00 | 0.00 | 0.00 | 1 | 0.00 | 0.00 | 0.00 | 0.00 | 0.67 | * |
| 216 | Porphyromonadaceae; g__ | 1 | 0.00 | 0.00 | 0.00 | 0.00 | 0.80 | 0 | 0.00 | 0.00 | 0.00 | 0.00 | 0.00 | * |
| 217 | Ruminococcaceae; g__ | 4 | 0.00 | 0.00 | 0.00 | 0.00 | 0.01 | 5 | 0.00 | 0.00 | 0.00 | 0.00 | 0.92 | * |
| 218 | Ruminococcaceae; g__ | 28 | 0.06 | 0.00 | 0.19 | 0.00 | 0.55 | 23 | 0.01 | 0.00 | 0.04 | 0.00 | 0.91 | 0.081 |
| 219 | Clostridia; o__; f__; g__ | 6 | 0.00 | 0.00 | 0.00 | 0.00 | 0.76 | 2 | 0.00 | 0.00 | 0.00 | 0.00 | 0.36 | * |
| 220 | Prevotellaceae; g__ | 2 | 0.00 | 0.00 | 0.00 | 0.00 | 0.45 | 2 | 0.00 | 0.00 | 0.00 | 0.00 | 0.86 | * |
| 221 | Butyricicoccus | 41 | 0.14 | 0.05 | 0.38 | 0.00 | 0.77 | 32 | 0.18 | 0.06 | 0.41 | 0.00 | 1.05 | 0.349 |
| 222 | Prevotella | 2 | 0.00 | 0.00 | 0.00 | 0.00 | 0.93 | 0 | 0.00 | 0.00 | 0.00 | 0.00 | 0.00 | * |
| 223 | Ruminococcaceae; g__ | 2 | 0.00 | 0.00 | 0.00 | 0.00 | 0.71 | 1 | 0.00 | 0.00 | 0.00 | 0.00 | 0.01 | * |
| 224 | Clostridium XlVb | 20 | 0.00 | 0.00 | 0.15 | 0.00 | 0.87 | 12 | 0.00 | 0.00 | 0.13 | 0.00 | 0.52 | 0.369 |
| 225 | Ruminococcaceae; g__ | 22 | 0.01 | 0.00 | 0.05 | 0.00 | 0.73 | 17 | 0.00 | 0.00 | 0.07 | 0.00 | 0.24 | 0.926 |
| 226 | unknown | 8 | 0.00 | 0.00 | 0.00 | 0.00 | 0.38 | 4 | 0.00 | 0.00 | 0.00 | 0.00 | 0.92 | * |
| 227 | Clostridium XlVb | 13 | 0.00 | 0.00 | 0.03 | 0.00 | 0.63 | 14 | 0.00 | 0.00 | 0.05 | 0.00 | 0.29 | 0.689 |
| 228 | unknown | 2 | 0.00 | 0.00 | 0.00 | 0.00 | 0.90 | 3 | 0.00 | 0.00 | 0.00 | 0.00 | 0.30 | * |
| 229 | Bacteroidetes; c__; o__; f__; g__ | 1 | 0.00 | 0.00 | 0.00 | 0.00 | 0.59 | 0 | 0.00 | 0.00 | 0.00 | 0.00 | 0.00 | * |
| 230 | Lachnospiraceae; g__ | 11 | 0.00 | 0.00 | 0.01 | 0.00 | 0.50 | 7 | 0.00 | 0.00 | 0.00 | 0.00 | 0.72 | 0.710 |
| 231 | unknown | 3 | 0.00 | 0.00 | 0.00 | 0.00 | 0.88 | 1 | 0.00 | 0.00 | 0.00 | 0.00 | 0.00 | * |
| 232 | Ruminococcaceae; g__ | 19 | 0.00 | 0.00 | 0.01 | 0.00 | 0.43 | 23 | 0.01 | 0.00 | 0.03 | 0.00 | 0.86 | 0.054 |
| 234 | unknown | 1 | 0.00 | 0.00 | 0.00 | 0.00 | 0.32 | 1 | 0.00 | 0.00 | 0.00 | 0.00 | 0.73 | * |
| 235 | Clostridiales; f__; g__ | 10 | 0.00 | 0.00 | 0.00 | 0.00 | 0.66 | 9 | 0.00 | 0.00 | 0.02 | 0.00 | 1.05 | 0.617 |
| 236 | unknown | 0 | 0.00 | 0.00 | 0.00 | 0.00 | 0.00 | 1 | 0.00 | 0.00 | 0.00 | 0.00 | 0.70 | * |
| 237 | Lachnospiracea_incertae_sedis | 36 | 0.04 | 0.01 | 0.09 | 0.00 | 0.40 | 28 | 0.03 | 0.01 | 0.11 | 0.00 | 1.02 | 0.980 |
| 238 | Lachnospiraceae; g__ | 20 | 0.00 | 0.00 | 0.05 | 0.00 | 0.31 | 17 | 0.00 | 0.00 | 0.05 | 0.00 | 0.84 | 0.991 |
| 239 | Clostridium IV | 1 | 0.00 | 0.00 | 0.00 | 0.00 | 0.27 | 1 | 0.00 | 0.00 | 0.00 | 0.00 | 0.67 | * |
| 240 | unknown | 2 | 0.00 | 0.00 | 0.00 | 0.00 | 0.86 | 1 | 0.00 | 0.00 | 0.00 | 0.00 | 0.25 | * |
| 241 | Victivallis | 13 | 0.00 | 0.00 | 0.02 | 0.00 | 0.46 | 13 | 0.00 | 0.00 | 0.05 | 0.00 | 0.78 | 0.532 |
| 242 | Parasutterella | 5 | 0.00 | 0.00 | 0.00 | 0.00 | 0.83 | 0 | 0.00 | 0.00 | 0.00 | 0.00 | 0.00 | * |
| 243 | Porphyromonadaceae; g__ | 14 | 0.00 | 0.00 | 0.03 | 0.00 | 0.75 | 23 | 0.02 | 0.00 | 0.05 | 0.00 | 0.27 | 0.016 |
| 246 | unknown | 1 | 0.00 | 0.00 | 0.00 | 0.00 | 0.04 | 1 | 0.00 | 0.00 | 0.00 | 0.00 | 0.64 | * |
| 247 | Clostridiales; f__; g__ | 12 | 0.00 | 0.00 | 0.01 | 0.00 | 0.56 | 2 | 0.00 | 0.00 | 0.00 | 0.00 | 0.11 | * |
| 249 | Collinsella | 2 | 0.00 | 0.00 | 0.00 | 0.00 | 0.58 | 1 | 0.00 | 0.00 | 0.00 | 0.00 | 0.03 | * |
| 250 | unknown | 8 | 0.00 | 0.00 | 0.00 | 0.00 | 0.60 | 5 | 0.00 | 0.00 | 0.00 | 0.00 | 0.17 | 0.319 |
| 251 | unknown | 8 | 0.00 | 0.00 | 0.00 | 0.00 | 0.76 | 5 | 0.00 | 0.00 | 0.00 | 0.00 | 0.15 | 0.490 |
| 254 | unknown | 5 | 0.00 | 0.00 | 0.00 | 0.00 | 0.74 | 3 | 0.00 | 0.00 | 0.00 | 0.00 | 0.23 | * |
| 255 | Proteobacteria; c__; o__; f__; g__ | 4 | 0.00 | 0.00 | 0.00 | 0.00 | 0.58 | 1 | 0.00 | 0.00 | 0.00 | 0.00 | 0.02 | * |
| 256 | Bacteroidales; f__; g__ | 1 | 0.00 | 0.00 | 0.00 | 0.00 | 0.72 | 1 | 0.00 | 0.00 | 0.00 | 0.00 | 0.04 | * |
| 258 | Clostridium XI | 35 | 0.02 | 0.01 | 0.09 | 0.00 | 0.72 | 28 | 0.02 | 0.01 | 0.06 | 0.00 | 0.37 | 0.600 |
| 259 | Coprococcus | 31 | 0.07 | 0.00 | 0.10 | 0.00 | 0.39 | 22 | 0.05 | 0.00 | 0.15 | 0.00 | 0.73 | 0.739 |
| 261 | Ruminococcaceae; g__ | 34 | 0.06 | 0.01 | 0.08 | 0.00 | 0.40 | 34 | 0.09 | 0.04 | 0.17 | 0.00 | 0.74 | 0.012 |
| 263 | Firmicutes; c__; o__; f__; g__ | 7 | 0.00 | 0.00 | 0.00 | 0.00 | 0.70 | 1 | 0.00 | 0.00 | 0.00 | 0.00 | 0.03 | * |
| 265 | Methanobrevibacter | 9 | 0.00 | 0.00 | 0.00 | 0.00 | 0.28 | 6 | 0.00 | 0.00 | 0.00 | 0.00 | 0.63 | 0.830 |
| 266 | unknown | 0 | 0.00 | 0.00 | 0.00 | 0.00 | 0.00 | 1 | 0.00 | 0.00 | 0.00 | 0.00 | 0.55 | * |
| 268 | Clostridium sensu stricto | 16 | 0.00 | 0.00 | 0.03 | 0.00 | 0.67 | 18 | 0.00 | 0.00 | 0.03 | 0.00 | 0.33 | 0.578 |
| 271 | Bacteroidetes; c__; o__; f__; g__ | 6 | 0.00 | 0.00 | 0.00 | 0.00 | 0.11 | 8 | 0.00 | 0.00 | 0.00 | 0.00 | 0.69 | 0.196 |
| 273 | Succiniclasticum | 1 | 0.00 | 0.00 | 0.00 | 0.00 | 0.61 | 0 | 0.00 | 0.00 | 0.00 | 0.00 | 0.00 | * |
| 274 | Butyricimonas | 18 | 0.00 | 0.00 | 0.10 | 0.00 | 0.57 | 18 | 0.01 | 0.00 | 0.17 | 0.00 | 0.46 | 0.484 |
| 275 | Clostridiales; f__; g__ | 6 | 0.00 | 0.00 | 0.00 | 0.00 | 0.27 | 7 | 0.00 | 0.00 | 0.00 | 0.00 | 0.64 | 0.515 |
| 277 | Lachnospiraceae; g__ | 33 | 0.02 | 0.00 | 0.05 | 0.00 | 0.35 | 32 | 0.03 | 0.02 | 0.06 | 0.00 | 0.86 | 0.114 |
| 279 | unknown | 4 | 0.00 | 0.00 | 0.00 | 0.00 | 0.62 | 1 | 0.00 | 0.00 | 0.00 | 0.00 | 0.23 | * |
| 281 | unknown | 7 | 0.00 | 0.00 | 0.00 | 0.00 | 0.61 | 2 | 0.00 | 0.00 | 0.00 | 0.00 | 0.29 | * |
| 282 | Allobaculum | 18 | 0.00 | 0.00 | 0.01 | 0.00 | 0.52 | 10 | 0.00 | 0.00 | 0.00 | 0.00 | 0.57 | 0.330 |
| 284 | Ruminococcaceae; g__ | 11 | 0.00 | 0.00 | 0.01 | 0.00 | 0.56 | 15 | 0.00 | 0.00 | 0.04 | 0.00 | 0.72 | 0.145 |
| 292 | Clostridiales; f__; g__ | 3 | 0.00 | 0.00 | 0.00 | 0.00 | 0.22 | 6 | 0.00 | 0.00 | 0.00 | 0.00 | 0.75 | * |
| 302 | Allisonella | 7 | 0.00 | 0.00 | 0.00 | 0.00 | 0.16 | 5 | 0.00 | 0.00 | 0.00 | 0.00 | 0.55 | 0.935 |
| 303 | unknown | 19 | 0.00 | 0.00 | 0.04 | 0.00 | 0.63 | 11 | 0.00 | 0.00 | 0.02 | 0.00 | 0.17 | 0.207 |
| 309 | Lachnospiraceae; g__ | 38 | 0.13 | 0.07 | 0.27 | 0.00 | 0.71 | 30 | 0.12 | 0.04 | 0.30 | 0.00 | 0.69 | 0.678 |
| 311 | Eubacterium | 2 | 0.00 | 0.00 | 0.00 | 0.00 | 0.15 | 4 | 0.00 | 0.00 | 0.00 | 0.00 | 0.60 | * |
| 312 | Lachnospiraceae; g__ | 7 | 0.00 | 0.00 | 0.00 | 0.00 | 0.59 | 6 | 0.00 | 0.00 | 0.00 | 0.00 | 0.29 | 0.969 |
| 336 | Ruminococcaceae; g__ | 32 | 0.02 | 0.01 | 0.07 | 0.00 | 0.57 | 28 | 0.05 | 0.01 | 0.10 | 0.00 | 0.71 | 0.211 |
| 363 | Porphyromonadaceae; g__ | 1 | 0.00 | 0.00 | 0.00 | 0.00 | 0.66 | 0 | 0.00 | 0.00 | 0.00 | 0.00 | 0.00 | * |
| 382 | Ruminococcaceae; g__ | 20 | 0.00 | 0.00 | 0.11 | 0.00 | 2.05 | 15 | 0.00 | 0.00 | 0.07 | 0.00 | 2.49 | 0.685 |
| 394 | Firmicutes; c__; o__; f__; g__ | 4 | 0.00 | 0.00 | 0.00 | 0.00 | 0.28 | 4 | 0.00 | 0.00 | 0.00 | 0.00 | 1.58 | * |
| 417 | Alistipes | 14 | 0.00 | 0.00 | 0.02 | 0.00 | 0.94 | 18 | 0.01 | 0.00 | 0.05 | 0.00 | 1.49 | 0.185 |
| 591 | Lachnospiraceae; g__ | 37 | 0.17 | 0.09 | 0.32 | 0.00 | 1.69 | 34 | 0.25 | 0.15 | 0.39 | 0.00 | 1.37 | 0.161 |
| 659 | Lachnospiraceae; g__ | 30 | 0.03 | 0.00 | 0.10 | 0.00 | 0.47 | 25 | 0.05 | 0.00 | 0.16 | 0.00 | 0.80 | 0.872 |
| 730 | Streptococcus | 36 | 0.02 | 0.01 | 0.05 | 0.00 | 0.24 | 30 | 0.03 | 0.01 | 0.04 | 0.00 | 0.78 | 0.857 |
| 828 | Megasphaera | 1 | 0.00 | 0.00 | 0.00 | 0.00 | 1.90 | 0 | 0.00 | 0.00 | 0.00 | 0.00 | 0.00 | * |
| 847 | Clostridiales; f__; g__ | 3 | 0.00 | 0.00 | 0.00 | 0.00 | 0.30 | 2 | 0.00 | 0.00 | 0.00 | 0.00 | 2.59 | * |
| 921 | Lachnospiraceae; g__ | 36 | 0.12 | 0.04 | 0.21 | 0.00 | 1.70 | 28 | 0.13 | 0.06 | 0.27 | 0.00 | 1.98 | 0.955 |
| 985 | Roseburia | 42 | 2.54 | 1.53 | 5.10 | 0.01 | 15.82 | 35 | 2.57 | 1.35 | 5.26 | 0.02 | 10.81 | 0.898 |
| 990 | Blautia | 7 | 0.00 | 0.00 | 0.00 | 0.00 | 0.15 | 7 | 0.00 | 0.00 | 0.00 | 0.00 | 0.61 | 0.327 |
| 1007 | Bacteroides | 9 | 0.00 | 0.00 | 0.00 | 0.00 | 0.31 | 9 | 0.00 | 0.00 | 0.01 | 0.00 | 2.45 | 0.499 |
| 1048 | Akkermansia | 5 | 0.00 | 0.00 | 0.00 | 0.00 | 2.03 | 8 | 0.00 | 0.00 | 0.00 | 0.00 | 30.83 | 0.207 |
| 1052 | Blautia | 41 | 0.23 | 0.11 | 0.46 | 0.00 | 1.61 | 34 | 0.38 | 0.22 | 0.75 | 0.00 | 1.47 | 0.024 |
| 1059 | Lachnospiraceae; g__ | 12 | 0.00 | 0.00 | 0.01 | 0.00 | 0.32 | 17 | 0.00 | 0.00 | 0.02 | 0.00 | 1.10 | 0.191 |
| 1061 | Alistipes | 35 | 0.24 | 0.02 | 0.74 | 0.00 | 3.28 | 34 | 0.59 | 0.16 | 1.08 | 0.00 | 3.54 | 0.021 |
| 1068 | Parabacteroides | 35 | 0.03 | 0.01 | 0.18 | 0.00 | 0.52 | 26 | 0.04 | 0.00 | 0.14 | 0.00 | 0.85 | 0.934 |
| 1071 | Barnesiella | 24 | 0.01 | 0.00 | 0.45 | 0.00 | 3.95 | 22 | 0.13 | 0.00 | 0.69 | 0.00 | 4.02 | 0.337 |
| 1090 | Lachnospiraceae; g__ | 3 | 0.00 | 0.00 | 0.00 | 0.00 | 0.04 | 5 | 0.00 | 0.00 | 0.00 | 0.00 | 0.69 | * |
| 1093 | Prevotella | 2 | 0.00 | 0.00 | 0.00 | 0.00 | 2.83 | 0 | 0.00 | 0.00 | 0.00 | 0.00 | 0.00 | * |
| 1104 | Butyricimonas | 22 | 0.01 | 0.00 | 0.10 | 0.00 | 0.70 | 16 | 0.00 | 0.00 | 0.08 | 0.00 | 0.27 | 0.645 |
| 1132 | Lachnospiraceae; g__ | 8 | 0.00 | 0.00 | 0.00 | 0.00 | 1.87 | 4 | 0.00 | 0.00 | 0.00 | 0.00 | 0.08 | * |
| 1138 | Bacteroides | 3 | 0.00 | 0.00 | 0.00 | 0.00 | 2.21 | 1 | 0.00 | 0.00 | 0.00 | 0.00 | 1.87 | * |
| 1139 | Dialister | 21 | 0.00 | 0.00 | 0.05 | 0.00 | 6.16 | 10 | 0.00 | 0.00 | 0.01 | 0.00 | 2.34 | 0.066 |
| 1141 | Prevotella | 12 | 0.00 | 0.00 | 0.10 | 0.00 | 3.65 | 2 | 0.00 | 0.00 | 0.00 | 0.00 | 0.66 | * |
| 1153 | Lachnospiraceae; g__ | 20 | 0.00 | 0.00 | 0.21 | 0.00 | 0.69 | 13 | 0.00 | 0.00 | 0.10 | 0.00 | 3.28 | 0.391 |
| 1173 | Ruminococcaceae; g__ | 33 | 0.18 | 0.01 | 0.84 | 0.00 | 3.88 | 29 | 0.05 | 0.01 | 0.38 | 0.00 | 4.78 | 0.461 |
| 1180 | Lachnospiraceae; g__ | 29 | 0.05 | 0.00 | 0.18 | 0.00 | 0.97 | 21 | 0.02 | 0.00 | 0.16 | 0.00 | 0.49 | 0.480 |
| 1185 | Dialister | 2 | 0.00 | 0.00 | 0.00 | 0.00 | 3.90 | 0 | 0.00 | 0.00 | 0.00 | 0.00 | 0.00 | * |
| 1188 | Alistipes | 12 | 0.00 | 0.00 | 0.03 | 0.00 | 0.28 | 12 | 0.00 | 0.00 | 0.09 | 0.00 | 2.12 | 0.574 |
| 1189 | Bifidobacterium | 20 | 0.00 | 0.00 | 0.04 | 0.00 | 0.49 | 23 | 0.03 | 0.00 | 0.10 | 0.00 | 0.86 | 0.118 |
| 1214 | Bacteroides | 38 | 0.39 | 0.08 | 0.76 | 0.00 | 1.77 | 35 | 0.34 | 0.20 | 0.86 | 0.01 | 3.06 | 0.616 |
| 1215 | Prevotella | 12 | 0.00 | 0.00 | 0.01 | 0.00 | 10.44 | 4 | 0.00 | 0.00 | 0.00 | 0.00 | 0.11 | * |

* p-values were not adjusted and p-values were not calculated when an OTU was present in less than 5 individuals in one of the two groups. c__; class unknown. o__; order unknown. f__; family unknown. g__; genus unknown.
